# AtIAR1 is a Zn transporter that regulates auxin metabolism in *Arabidopsis thaliana*

**DOI:** 10.1093/jxb/erad468

**Published:** 2023-11-21

**Authors:** Thomas Gate, Lionel Hill, Anthony J Miller, Dale Sanders

**Affiliations:** Biochemistry and Metabolism, John Innes Centre, Norwich NR4 7UH, UK; Biochemistry and Metabolism, John Innes Centre, Norwich NR4 7UH, UK; Biochemistry and Metabolism, John Innes Centre, Norwich NR4 7UH, UK; Biochemistry and Metabolism, John Innes Centre, Norwich NR4 7UH, UK; University of Warwick, UK

**Keywords:** Auxin, endoplasmic reticulum, hormone conjugation, IARI, membrane transport, zinc

## Abstract

Root growth in Arabidopsis is inhibited by exogenous auxin–amino acid conjugates, and mutants resistant to one such conjugate [indole-3-acetic acid (IAA)–Ala] map to a gene (*AtIAR1*) that is a member of a metal transporter family. Here, we test the hypothesis that AtIAR1 controls the hydrolysis of stored conjugated auxin to free auxin through zinc transport. *AtIAR1* complements a yeast mutant sensitive to zinc, but not manganese- or iron-sensitive mutants, and the transporter is predicted to be localized to the endoplasmic reticulum/Golgi in plants. A previously identified *Atiar1* mutant and a non-expressed T-DNA mutant both exhibit altered auxin metabolism, including decreased IAA–glucose conjugate levels in zinc-deficient conditions and insensitivity to the growth effect of exogenous IAA–Ala conjugates. At a high concentration of zinc, wild-type plants show a novel enhanced response to root growth inhibition by exogenous IAA–Ala which is disrupted in both *Atiar1* mutants. Furthermore, both *Atiar1* mutants show changes in auxin-related phenotypes, including lateral root density and hypocotyl length. The findings therefore suggest a role for AtIAR1 in controlling zinc release from the secretory system, where zinc homeostasis plays a key role in regulation of auxin metabolism and plant growth regulation.

## Teaser

Regulation of intracellular zinc compartmentation by IAR1 plays a key role in the control of auxin activity through zinc-sensitive release of IAA from IAA conjugate stores.

## Introduction

Auxin (indole-3-acetic acid, IAA) is a major plant hormone involved in many developmental processes in plants, including embryonic (Schiavone and Cooke, 1987; Liu *et al.*, 1993) and post-embryonic development through maintenance of the root apical meristem (Ioio *et al.,* 2008) and the shoot apical meristem (Reinhardt *et al*., 2000; Vernoux *et al*., 2011). Both polar transport and metabolism operate as major controls on auxin action.

Polar transport between and within cells and tissues is used to create concentration gradients which coordinate specific developmental patterns. In order to create auxin concentration gradients, various auxin transporters are required including the polarly localized long PINFORMED (PIN) auxin exporters (Okada *et al*., 1991; Friml *et al*., 2002). In addition, subcellular compartmentalization of IAA is also controlled by transporters. Transporters such as short PINs and PIN-LIKES (PILS) family members localize to the endoplasmic reticulum (ER) and coordinate IAA transport between the ER and cytosol (Mravec *et al*., 2009; Barbez *et al*., 2012; Simon *et al*., 2016).

The major routes for synthesis of IAA originate with Trp and then proceed either via indole-3-acetaldoxime (IAOx) and indole-3-acetonitrile (IAN) (Normanly *et al*., 1997; Hull *et al*., 2000; Mikkelsen *et al*., 2000) or via indole-3-pyruvic acid (IPA). The route via IPA involves TAA/TAR and YUCCA enzymes (Mashiguchi *et al*., 2011; Stepanova *et al*., 2011; Won *et al*., 2011), and severe developmental phenotypes in mutants suggest that the pathway involving YUCCA is the major pathway to synthesize IAA (Cheng *et al*., 2007; Stepanova *et al*., 2008).

The concentration of active IAA is then also controlled through its conjugation into inactive storage forms and degradation via oxidation (Mellor *et al*., 2016). The role and importance of conjugates in IAA concentration modulation and auxin action is currently poorly understood. These inactive conjugates can be irreversibly or reversibly formed. Irreversibly formed conjugates include acidic amino acid-linked IAAs such as IAA–Asp and IAA–Glu (Östin *et al*., 1998) formed from IAA through the action of enzymes in the Gretchen Hagen 3 (GH3) family (Staswick *et al*., 2005; Di Mambo *et al*., 2019). Reversibly formed conjugates are hypothesized to act as a temporary inactive reservoir of IAA, and include ester-linked IAAs such as IAA–glucose (IAA–Glc) (Jackson *et al*., 2001; Jin *et al*., 2013), methylated IAA (Qin *et al*., 2005; Yang *et al*., 2008), and some IAA–amino acid conjugates (IAA–Ala, IAA–Leu, and IAA–Phe among others). In the case of these non-acidic IAA–amino acids, the conjugation is reversible through the activity of IAA–amino acid amidohydrolases (Bartel and Fink, 1995; Davies *et al*., 1999; LeClere *et al.*, 2002; Sanchez Carranza *et al*., 2016).

In Arabidopsis, IAA–amino acid hydrolysis to IAA is governed by four of a seven-member Ilr1-like amidohydrolase family (LeClere *et al*., 2002). The four enzymes shown to have IAA–amino acid hydrolysis activity are IAA-LEUCINE RESISTANT 1 (ILR1) (Bartel and Fink, 1995), ILR-LIKE 1 (ILL1), ILL2, and IAA-ALANINE RESISTANT 3 (IAR3) (Davies *et al*., 1999), all of which show enhanced *in vitro* activity with increased Mn and Co and are inhibited by Zn ions (LeClere *et al*., 2002). The IAA–amino acid-hydrolysing enzymes were all determined to be localized to the ER through bioinformatic, proteomic, and green fluorescent protein (GFP) tagging analysis (Bartel and Fink, 1995; Davies *et al*., 1999; LeClere *et al.,* 2002; Sanchez Carranza *et al*., 2016).

High concentrations of IAA conjugates elicit a characteristic shortening of root lengths in wild-type plants (Bartel and Fink, 1995; Lasswell *et al*., 2000). To identify genes that control auxin conjugate hydrolysis, screening conditions were therefore established with high concentrations of IAA–amino acid conjugates to find mutants insensitive to the effects of IAA conjugates (Bartel and Fink, 1995; Lasswell *et al*., 2000). Among many mutants identified, these screens revealed genes involved in metal homeostasis, such as *IAA-Leu resistance 2* (*AtILR2*) which when mutated increased Mn sequestration (Magidin *et al*., 2003), and *AtILR3* (Rampey *et al*., 2006) which is involved in Fe deficiency (Li *et al*., 2016) and Fe excess responses (Tissot *et al*., 2019). Mutants insensitive to IAA–Ala resulted in identification of the *IAA-Ala resistance1* (*AtIAR1*) gene (Lasswell *et al*., 2000). Seven *Atiar1* mutants with a range of phenotypic severity were characterized. The *Atiar1* mutants were all shown to be less sensitive to all IAA–amino acid conjugates tested including not only IAA–Ala, but also IAA–Leu and IAA–Phe, among others. High auxin *Atsuperroot1* plants (Boerjan *et al*., 1995; Celenza *et al*., 1995) had their high auxin phenotype of reduced root growth partially suppressed after 10 d in *Atsuperroot1 Atiar1* double mutants, suggesting that *Atiar1* mutants were low in auxin (Lasswell *et al*., 2000).

Evidence available at the time of the discovery of *AtIAR1* and subsequently has indicated that ion homeostasis might play a role in IAA–Ala insensitivity of *Atiar1* mutants. Increasing the Mn content of the media gradually suppressed this IAA–amino acid insensitivity in *Atiar1* plants (Lasswell *et al*., 2000). *AtIAR1* was identified as a member of the ZRT IRT1-related protein (ZIP) transporter family, and expression of the *AtIAR1* mouse homologue *MmKE4* (*MmZIP7*) complemented the *Atiar1* phenotype (Lasswell *et al*., 2000). Further work has shown that MmZIP7 functions as a Golgi-localized Zn transporter in mice (Huang *et al*., 2005), suggesting that the *Atiar1* phenotype is metal transport related.

In addition, unlike the *Atiar3* and *Atilr1* hydrolase mutants, the *Atiar1* phenotype was partially compensated by mutations in the *METAL TOLERANCE PROTEIN 5* gene (*AtMTP5*) (Rampey *et al*., 2013). AtMTP5 was predicted and later established (Fujiwara *et al*., 2015) to act as a cytoplasmic Zn exporter, which is hypothesized to act antagonistically to AtIAR1 in the secretory pathway to control the activity of IAA–amino acid amidohydrolases (Rampey *et al*., 2013).

AtIAR1 is therefore thought to play a key role in controlling hydrolysis of inactive auxin conjugates, and hence buffering of free auxin. However, the physiological significance of this control, the metal specificity of AtIAR1, and the relationship of AtIAR1 to auxin metabolism and auxin-related phenotypes remains to be established. Here, we investigate these relationships and show a major role for AtIAR1 in linking Zn homeostasis to regulation of auxin activity.

## Materials and methods

### Phylogenetic analysis of the ZIP transporter family

To determine the relationship of AtIAR1 to other plant ZIPs as well as to the wider ZIP transporter family, a phylogenetic tree was constructed. Amino acid sequences of ZIP proteins were gathered using the tBLASTn function on the BLAST server (Boratyn *et al*., 2013) using the amino acid sequences of AtIAR1 (Uniprot accession no. Q9M647) and AtIRT1 (Uniprot accession no. Q38856) as the queries and collecting sequences from organisms within animal (*Drosophila melanogaster* and *Homo sapiens*), plant (*Arabidopsis thaliana*, *Oryza sativa*, and *Medicago truncatula*) and fungal (*Saccharomyces cerevisiae*) kingdoms. These sequences were aligned and phylogeny calculated within the MEGA-X software (Kumar *et al*., 2018). For alignment, MUSCLE (Edgar, 2004) was used, utilizing default settings and phylogeny calculated with the Maximum Likelihood method and the Jones–Taylor–Thornton (JTT) matrix-based model (Jones *et al*., 1992) with a gamma distribution of five categories to model different evolutionary rates at different sites. The phylogenetic tree was then constructed from 100 bootstrap replicates and initial trees using BioNJ and Neighbor–Joining algorithms on the JTT-generated matrix.

### *Saccharomyces cerevisiae* strains and constructs for expression

AtIAR1 (*Arabidopsis thaliana* IAA-Ala resistant 1) functionality including transport capabilities was assessed by transforming *AtIAR1*-based constructs into *S. cerevisiae* transporter mutants using BY4741 and DY1457 strains as controls as appropriate. Details of these mutants are shown in Table 1.

**Table 1.** List of *Saccharomyces cerevisiae* strains used in this study

Strain	Description	Genotype	Source
BY4741	Background for *smf1*Δ	*MATa*; *his3*Δ1; *leu2*Δ0; *met15*Δ0; *ura3*Δ0	Podar *et al*. (2012)
*smf1*Δ	Mn import mutant	BY4741; *MATa*; *his3*Δ1; *leu2*Δ0; *met15*Δ0; *ura3*Δ0; *YOL122c*::*kanMX4*	Euroscarf
DY1457	Background for *zrt1*Δ *zrt2*Δ and *fet3*Δ *fet4*Δ	DY1457; *MATa*; *ade1*/+; *can1*; *his3*; *leu2*; *trp1*; *ura3*	Evens *et al*. (2017)
*zrt1*Δ *zrt2*Δ	Zn import mutant	DY1457; *MATa*; *ade1*/+; *can1*; *his3*; *leu2*; *trp1*; *ura3*; *zrt1*::*LEU2*, *zrt2*::*HIS3*	Evens *et al*. (2017)
*fet3*Δ *fet4*Δ	Fe import mutant	DY1457; *MATa*; *ade1*/+; *can1*; *his3*; *leu2*; *trp1*; *ura3*; *fet3-2*::*HIS3*, *fet3-1*::*LEU2*	Evens *et al*. (2017)

To assess the function of AtIAR1, constructs for heterologous expression of *AtIAR1* in *S. cerevisiae* were assembled into a pYES2 (ThermoFisher, V82520) vector, allowing for selection in bacterial and yeast hosts with ampicillin resistance and uracil autotrophy, respectively. Constructs were synthezised (Integrated DNA Technologies) to represent codon-optimized *AtIAR1* cDNA from the *A. thaliana* Col-0 ecotype as well as for the *Atiar1-3* mutant identified by Lasswell *et al*. (2000).

### *Saccharomyces cerevisiae* growth and transformation

Growth of non-transformed *S. cerevisiae* strains was conducted in YPAD medium with or without agar. YPAD medium consisted of 1% (w/v) yeast extract (Millipore, 70161), 2% (w/v) peptone (Formedium, PEP02), 2% (w/v) glucose (Fisher Scientific, G/0500/53), 0.002% (w/v) adenine (Sigma-Aldrich, A2786), and 2% (w/v) agar (Formedium, AGA03) if required.

Once transformed with recombinant plasmids, *S. cerevisiae* gained uracil autotrophy and were grown on synthetic complete medium lacking uracil (SC-U). In addition, expression of the *AtIAR1-*based construct was controlled by glucose or galactose addition. Unless otherwise stated, SC-U was made using glucose to repress expression of the *AtIAR1*-based construct. SC-U medium consisted of 0.69% (w/v) yeast nitrogen base (Formedium, CYN0405), 2% (w/v) glucose or galactose (Formedium, GAL03), 0.19% (w/v) Kaiser mixture synthetic complete uracil drop-out (Formedium, DSCK102), and 2% (w/v) agar if required. For *fet3*Δ *fet4*Δ transformants, the pH of SC-U liquid and solid media was set to 4.5 because of slow growth, whilst all other strains were grown at pH 5.3.

Non-transformed *S. cerevisiae* strains were streaked onto solid YPAD plates and incubated at 28 °C for 2–4 d until individual colonies were visible. These strains were then transformed using the lithium acetate method (Gietz and Wood, 2002). Transformed cells were then grown on SC-U selective plates for 2–4 d at 30 °C, with colonies replated and confirmed by PCR.

### *Saccharomyces cerevisiae* drop assays

To assay complementation of mutant strains on restrictive media, a drop assay was performed with the transformed *S. cerevisiae* strains. Individual colonies of the transformed *S. cerevisiae* were grown in 10 ml of liquid SC-U overnight at 30 °C, shaking at 210 rpm. These cultures were pelleted by centrifugation at 1500 *g* for 10 min at room temperature, with the supernatant discarded and the pellet resuspended in dH_2_O. After repeating the centrifugation step and discarding the supernatant, the pellet was then resuspended in dH_2_O to an OD_600_ of 1.0. A 10 µl aliquot of this diluted culture and serial dilutions up to 10^5^ were then pipetted onto SC-U agar plates containing 2% (w/v) galactose to induce expression of the *AtIAR1*-based construct.

Restrictive media for transformants were based on those from previous publications (Podar *et al*., 2012; Milner *et al*., 2013), with some minor alterations as detailed below. The *smf1*Δ strain was sensitive to Mn deficiency, and complementation therefore was assayed in medium with 20 mM EGTA (Millipore, 324626) and 50 mM MES (Sigma-Aldrich, M8250) at pH 5.5. The *zrt1*Δ *zrt2*Δ strain was sensitive to Zn deficiency, and complementation was therefore assayed in medium with 500 µM ZnSO_4_ (Sigma-Aldrich, Z1001) and 1 mM EDTA (Sigma-Aldrich, E9884). The *fet3*Δ *fet4*Δ strain was sensitive to Fe deficiency, and complementation was therefore assayed in medium with 25 µM bathophenanthroline disulfonate (BPDS; Sigma-Aldrich, 146617) and 20 mM MES.

### Plant materials

To assess the impact of loss of AtIAR1 activity in *A. thaliana*, two different mutant lines were sourced. The *Atiar1-3* mutant used by Lasswell *et al*. (2000) was kindly provided by the Bartel lab. The second mutant was a T-DNA insertion mutant (Alonso *et al*., 2003) (SALK_ 047876C, obtained through the Nottingham Arabidopsis Stock Centre) in a Col-0 background, hereafter referred to as *Atiar1-t*. The *A. thaliana* Col-0 ecotype was therefore used as a wild-type reference for both these mutants. Genotyping of the *Atiar1-3* plants was conducted as described by Lasswell *et al*. (2000) whilst genotyping of the *Atiar1-t* plants was conducted using left border primer GTCTCTTGCGTGAATGAGAGG, right border primer CATTTCTGCAAGAACTCCAGC, and T-DNA border primer ATTTTGCCGATTTCGGAAC.

### Arabidopsis seed sterilization and vernalization

*Arabidopsis thaliana* seeds were surface-sterilized using 70% (v/v) ethanol followed by treatment with 5% (v/v) bleach and 0.05% (v/v) Tween-20 (Sigma-Aldrich, P2287) solution for 10 min. After three 10 min washes with dH_2_O, the seeds were placed into sterile 0.1% (w/v) agarose (Melford Biolaboratories Ltd, A20080) solutions. Sterilized seeds were then vernalized for 48 h at 4 °C in darkness, before being placed into the relevant medium.

### EDTA-washed Hoagland’s medium-based agar

In order to create Zn-deficient conditions on solid media, agar was washed with EDTA as described in Sinclair *et al*. (2018). For this process, 30 g of agar was washed three times with 1 litre of 50 mM EDTA pH 8.0, twice for 5 h then once for 14 h. The agar was then washed again with 1 litre of dH_2_O four times for 4 h then once for 14 h. To maintain suspension of agar during washes, the agar solution was shaken at 130 rpm. To exchange media, the agar suspension was filtered through Miracloth (475855, Merck Millipore Ltd, Watford, UK). After the water washes, the agar was air-dried on filter paper (185 mm diameter, FT3205185, Sartorius Ltd, UK) for 1 d at room temperature, before adding the modified Hoagland growth medium (Hoagland and Arnon, 1938) (Supplementary Table S1) with 0, 1, or 150 µM Zn and sucrose (Formedium, S/8600/60) to 1% (w/v) to yield medium with 1.5% (w/v) agar, with pH set to 5.7 using KOH.

### Root and shoot growth phenotyping

To assess phenotypes, sterilized and vernalized seeds were sown on Hoagland medium-based EDTA-washed agar media with 0, 1, or 150 µM ZnSO_4_ and with either no auxin, 100 nM IAA, or 20 µM IAA–Ala added, and grown vertically. After 10 d, images of plants were taken for measurement of primary root length, shoot hypocotyl length, and lateral root (LR) density. For calculating the percentage change in auxin-containing medium, the phenotypic measurements were compared with the mean measurement in the same biological replicate grown on medium without any auxin added.

Skotomorphogenesis was induced as previously described by Mateo‐Bonmatí *et al*. (2021) by transferring vernalized seeds to light conditions at 21 °C for 8 h to allow germination, before transferring to darkness for an additional 4 d at 21 °C.

All images were analysed with Fiji (ImageJ) software.

### Plant metabolite quantification

To investigate the influence of Zn status and AtIAR1 activity on auxin metabolism, the abundance of several auxin-related metabolites was measured (Supplementary Table S2; Supplementary Fig. S1). These were chosen to represent precursors of IAA (Trp and IAN), IAA itself, and IAA conjugation and degradation products (IAA–Ala, IAA–Asp, IAA–Glc, and oxIAA).

Sterilized and vernalized seeds were sown on Hoagland solution-based EDTA-washed agar medium with 0, 1, or 150 µM ZnSO_4_, and grown vertically for 16 d. In a protocol adapted from Sugahara *et al*. (2020), 100 mg pools of 16-day-old whole seedlings were snap-frozen in liquid nitrogen and homogenized with 3 mm tungsten carbide beads with a TissueLyser LT homogenizer. A 333 µl aliquot of 80% (v/v) methanol containing the internal standard deuterated IAA (D_2_-IAA) at 500 nM was added to the homogenate, then centrifuged at 10 800 *g* for 1 min, and the supernatant was extracted and retained in a separate 2 ml Eppendorf vial on ice. Two further rounds of adding 333 µl of 80% (v/v) methanol, homogenization, and collecting supernatant were performed, resulting in 1 ml of 80% (v/v) methanol solution containing extracted metabolites and 166 nM D_2_-IAA.

The 80% (v/v) methanol solution was then subjected to liquid–liquid extraction using hexane. After adding 700 µl of hexane and inverting several times, the mixture was separated by centrifugation at 16 000 *g* for 3 min. The upper hexane layer was removed, and the hexane addition, inversion, and centrifugation steps repeated a further twice.

The resulting fraction was dried in a Genevac EZ-2 Elite Evaporator (SP Scientific), with a temperature limit of 45 °C, evaporation time of 90 min, and a drying time of 30 min. Dried samples were then resuspended in 100 µl of 80% (v/v) methanol containing no D_2_-IAA and passed through a 0.22 µm pore spin filter (8161, Corning Ltd).

### Liquid chromatography–mass spectrometry

Samples were analysed on an Acquity UPLC equipped with a Xevo TQ-S mass spectrometer (Waters). Separation was on a 100 × 2.1 mm 2.6μ Kinetex EVO C18 column (Phenomenex) using the following gradient of methanol (B) versus 0.5% formic acid in water (A), run at 0.4 ml min^–1^ and 40 °C: 0 min, 5% solvent B; 4 min, 90% B; 5 min, 90% B; 5.2 min, 5% B; 8 min, 5% B. Detection was by positive mode electrospray with spray chamber conditions of 1.5 kV capillary voltage, 500 °C desolvation gas temperature, 1000 l h^–1^ desolvation gas, 150 l h^–1^ cone gas, and a nebulizer pressure of 70 bar. Mass transitions and detection parameters for each analyte are shown in Supplementary Tables S3–S7. Analytes were quantified using internal standard calibration (D_2_-IAA as internal standard) using TargetLynx software (Waters) and are expressed in mol mg^–1^ FW.

### Expression analysis of AtIAR1

Whole 16-day-old seedlings were pooled into 100 mg samples which were snap-frozen in liquid nitrogen. The samples were then homogenized using 3 mm tungsten carbide beads and a TissueLyser LT homogenizer. RNA was extracted using an RNeasy Plant Mini Kit (74904, QIAGEN, Hilden, Germany) according to the manufacturer’s specifications, with RNA concentration measured with a NanoDrop™ 2000 spectrophotometer. To synthesize cDNA, a SuperScript™ IV First-Strand Synthesis System (Invitrogen, 18091050) was used according to the manufacturer’s specifications with each 20 µl reaction containing 4 µl of RNA (400 ng µl^–1^). Reactions were run at 37 °C for 50 min, then 70 °C for 15 min in a Mastercycler® pro thermocycler (Eppendorf UK Ltd, Stevenage, UK). cDNA was diluted 10-fold prior to use as a template in PCR. PCR was conducted using primers for cDNA of the full-length *AtIAR1* gene (ATGTCGTTCTCGCTGAGAAAG and TCATTCTATAAGAGAGATGCAAAGAG) and for cDNA of the *AtUBC* fragment (CTGCGACTCAGGGAATCTTCTAA and TTGTGCCATTGAATTGAACCC).

### Chlorophyll and shoot fresh weight analysis

Sterilized and vernalized seeds were sown onto Hoagland solution-based EDTA-washed agar medium with 0, 1, or 150 µM ZnSO_4_ and grown for 16 d before having the shoot fresh weight measured, and then were snap-frozen in 50 mg pools. These pools were homogenized using 3 mm tungsten carbide beads and the TissueLyser LT homogenizer. Samples were dissolved in 100% methanol (Fisher Scientific, 10674922) for 30 min at 40 °C, before being clarified by centrifugation at 10 000 *g* for 1 min. The resulting supernatant was then diluted as appropriate, and absorbances at 650 nm (*A*_650_) and 665 nm (*A*_665_) were measured using a spectrophotometer to calculate chlorophyll concentration based on the following equation (Porra *et al.*, 1989): Chl (µg ml^–1^)=22.5(*A*_650_)+4(*A*_665_)

Chlorophyl content was then reported as µg mg^–1^ FW.

## Results

### *AtIAR1* is a LIV-1 subfamily member, divergent from other Arabidopsis ZIP family genes

**Fig. 1.**
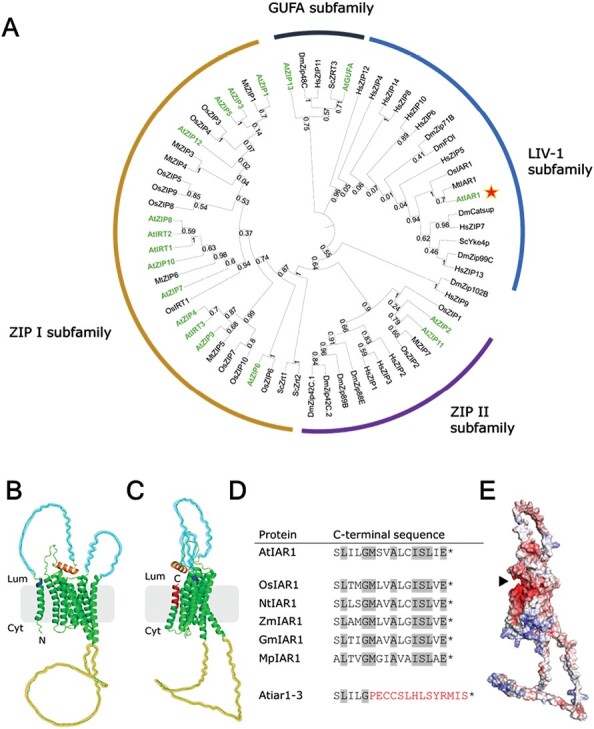
AtIAR1 phylogeny, structure, and *Atiar1* mutants used in this study. (A) Phylogenetic tree of ZIP family members. Sequences taken from within animal (*Drosophila melanogaster* and *Homo sapiens*), plant (*Arabidopsis thaliana* highlighted in green, *Oryza sativa*, and *Medicago truncatula*), and fungal (*Saccharomyces cerevisiae*) kingdoms. Numbers by branches show bootstrap support values. *AtIAR1* is indicated by the red star. (B) AlphaFold (Jumper *et al*., 2021) structural model of AtIAR1 oriented in the cytosol-down configuration and colour-coded by feature, with transmembrane helices in green, signal peptide cleavage site as predicted by signalP-5.0 (Almagro Armenteros *et al.*, 2019b) in dark blue, H-rich loops shaded light blue, acidic helix shaded orange, and ‘variable loop’ shaded grey. (C) AlphaFold structural model of AtIAR1, rotated from (B) and annotated similarly, with the addition of the red region representing the amino acids substituted in the Atiar1-3 variant of AtIAR1. (D) Amino acid sequences at the C-terminus in AtIAR1, AtIAR1 plant homologues, and Atiar1-3. Asterisks indicate STOP codons while the residues in red are those altered through a frameshift mutation in the *Atiar1-3* gene. Residues highlighted in grey are those showing 100% conservation in the sample of plant AtIAR1 homologues shown (*Oryza sativa*, *Nicotiana tabacum*, *Zea mays*, *Glycine max*, and *Marchantia polymorpha*). (E) Electrostatic surface of the AtIAR1 protein model rotated to the same degree of that in (C) generated by Adaptive Poisson–Boltzmann Solver (APBS) (Dolinsky *et al*., 2004), with red and blue corresponding to negatively charged and positively charged surfaces, respectively. The black triangle indicates the negatively charged pocket that might be disrupted in Atiar1-3.

The ZIP family can be split into four subfamilies (Jeong and Eide, 2013), with phylogenetic analysis showing that *AtIAR1* is the only *ZIP* gene in Arabidopsis found in the LIV-1 subfamily (Fig. 1A). The closest characterized homologues within this subfamily are human and mouse *ZIP7*, *DmCatsup*, and *ScYke4*, rather than the other *ZIP* genes found in Arabidopsis. Constitutive expression of the mouse homologue *MmZIP7* in *Atiar1* mutants restored IAA–Ala sensitivity (Lasswell *et al*., 2000), and in *ScYKE4* mutants restored calcofluor white resistance (Kumánovics *et al*., 2006), suggesting that AtIAR1 and its homologues possess a high degree of conservation of function across kingdoms.

The AlphaFold (Jumper *et al*., 2021) structural model of AtIAR1 predicts eight transmembrane helices in addition to a signal peptide, two histidine-rich loops on the luminal side of the membrane, and a large ‘variable loop’ on the cytosolic side found in many ZIP transporters (Guerinot, 2000) (Fig. 1B).

The illustrated topology of AtIAR1 is supported by immunofluorescence experiments placing the N-terminus (without signal peptide) of hZIP2 on the extracellular side of the membrane (Franz *et al*, 2014), by evidence of cytosolic metal binding and post-translational modifications on the cytosolic variable loop in AtIRT1 (Dubeaux *et al*, 2018), and by that of bioinformatics using the TOPCONS methodology (Tsirigos *et al*, 2015).

To investigate the role of AtIAR1, two different Arabidopsis mutants were used in this study, *Atiar1-t* and *Atiar1-3*. *Atiar1-t* is a T-DNA insertion line (SALK_ 047876C) not yet characterized, with the T-DNA insertion site within exon 10 encoding part of central helix 5. Therefore, *Atiar1-t* is predicted to be a full knockout which is confirmed by the lack of expression of full-length *AtIAR1* (Supplementary Fig. S2). By contrast, *Atiar1-3* is a partially characterized mutant found during a screen for IAA–Ala insensitivity by Lasswell *et al*. (2000) and contains a frameshift mutation, close to the 3' end of the coding region, which leads to changing only the final 11 amino acids (highlighted in red in Fig. 1C) whilst maintaining expression of a full-length *Atiar1-3* transcript (Supplementary Fig. S2). This C-terminus is highly conserved in plants (Fig. 1D) and may also play a role in AtIAR1 functionality by forming dimerization contacts as predicted through analysis of evolutionary coupling of residues in the ZIP family (Wiuf *et al*., 2022). The wild-type C-terminus is modelled to contribute to a negatively charged pocket on the luminal side of the membrane which might also contribute to activity (Fig. 1E).

### AtIAR1 transports Zn into the cytosol, possibly from the secretory system

In the model of AtIAR1 and AtMTP5 function proposed by Rampey *et al*. (2013), the metal transported by AtIAR1 was not directly assayed, although Zn was suspected, as evidenced by the metal transport capability of MTP proteins (Debrosses-Fonrouge *et al*., 2005), and later MTP5 itself in complex with MTP12 (Fujiwara *et al*., 2015). ZIP proteins in plants have previously been characterized as being capable of transporting Fe in the case of AtIRT1 (Korshunova *et al*., 1999) and Mn in the case of AtZIP2 (Milner *et al*., 2013) in addition to Zn ions. Complementation of yeast strains defective in metal import is a commonly used method for assessing the metal transport capabilities of plant ZIP proteins including from Arabidopsis (Milner *et al*., 2013). Therefore, a collection of strains defective in the import of Zn^2+^, Mn^2+^, and Fe^2+^ was transformed with *AtIAR1*-based constructs. The transformed strains were then assessed for their growth on Zn, Mn, or Fe restrictive media.

**Fig. 2.**
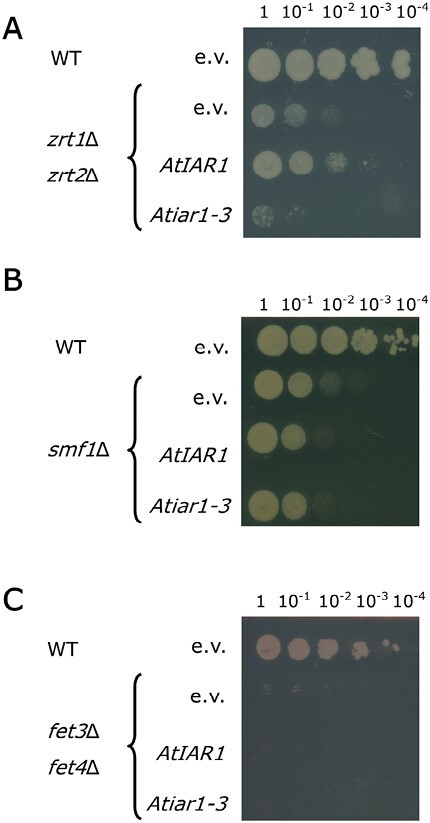
Complementation of metal import yeast mutants by *AtIAR1* constructs. Serial 10-fold dilutions (left to right) of transformed yeast cultures were pipetted onto restrictive media. Wild-type (WT) or mutant strains defective in the import of Zn (*zrt1*Δ *zrt2*Δ), Mn (*smf1*Δ), or Fe (*fet3*Δ *fet4*Δ) were transformed with empty vector (e.v.), *AtIAR1*, or *Atiar1-3* constructs. Transformed strains were then grown on (A) Zn-restrictive medium, (B) Mn-restrictive medium, or (C) Fe-restrictive medium.

As Fig. 2 shows, the *AtIAR1* construct lacks the ability to complement the Mn and Fe uptake mutants, while it is able to complement the Zn uptake mutant partially, implying that AtIAR1 shows specific transport activity for Zn as predicted. Transformation with the *Atiar1-3* construct did not lead to complementation, which could indicate either reduced localization to the plasma membrane in yeast or reduced transport activity of Atiar1-3 protein.

Several points of evidence indicate that AtIAR1 is localized to the secretory pathway in Arabidopsis. Hydrolases specific for IAA–amino acid conjugates such as IAA–Ala have been shown to localize to the ER, through bioinformatic, proteomic, and GFP tagging experiments (Bartel and Fink, 1995; Davies *et al*., 1999; LeClere *et al*., 2002; Sanchez Carranza *et al*., 2016). Since *Atiar1* mutants are insensitive to IAA–Ala (Lasswell *et al*., 2000), it is therefore likely that AtIAR1 is present at least on the ER membrane. In addition, the *Atiar1* phenotype was partially complemented by additional mutations in *AtMTP5* (Rampey *et al*., 2013). *AtMTP5* was found to encode a *cis*-Golgi-localized protein that interacts with AtMTP12 to facilitate transport of Zn into the Golgi (Fujiwara *et al*., 2015). Furthermore, expression of the AtIAR1 mouse homologue *MmKE4* (*MmZIP7*), which localizes to the ER/Golgi in mice (Taylor *et al*., 2004; Huang *et al*., 2005), complemented the *Atiar1* phenotype (Lasswell *et al*., 2000).

A range of software has been developed for predicting localization of proteins in plants. PProwler v1.2 (Hawkins and Bodén, 2006), TargetP v2.0 (Almagro Armenteros *et al.*, 2019a), and DeepLoc v2 (Thumuluri *et al*., 2022) were used in this study. All localization software tested showed that the most likely localization of AtIAR1 was in the ER or the secretory pathway (Table 2).

**Table 2.** AtIAR1 is predicted to localize to the secretory pathway

Prediction software	Subcellular prediction	Probability
PProwler v1.2	Secretory pathway	1.0
TargetP v2.0	Secretory pathway	0.923
DeepLoc v2.0	ER	0.648

Subcellular prediction with highest probability is listed along with the associated probability of this location.

Therefore, the current evidence suggests that AtIAR1 imports Zn to the cytosol from the secretory pathway, thereby playing a role in regulating Zn concentration within the lumen of the secretory pathway. The effect of the disruption of this transport activity in *Atiar1* plants on wider auxin metabolism, including that of conjugation and its relationship with media Zn levels, was therefore investigated.

### *Atiar1* mutants show disrupted wider auxin metabolism

Precise quantification of metabolite levels is essential to understanding changes in IAA metabolism. LC-MS was used to quantify IAA and IAA-related metabolites using D_2_-IAA as an internal standard based on previous approaches (Sugahara *et al*., 2020). A methodology adapted for the current study (Sugahara *et al*., 2020), as shown in the Materials and methods, produced an appropriate linear response range for metabolite analysis in Arabidopsis whole seedlings for a variety of IAA-related metabolites including IAA precursors (Trp and IAN), IAA, IAA conjugates (IAA–Ala, IAA–Asp, and IAA–Glc), and degradation pathway intermediates (oxIAA) (Supplementary Table S7). Levels of IAA–Ala were too low to detect in this analysis, a result that aligns with previous work suggesting that the content of IAA–Ala is below that of other conjugates including IAA–Glc and IAA–Asp (Sugahara *et al*., 2020). This differing abundance suggests some degree of selection between conjugates as an IAA store, although the mechanisms and significance of this selection remain unclear.

**Fig. 3.**
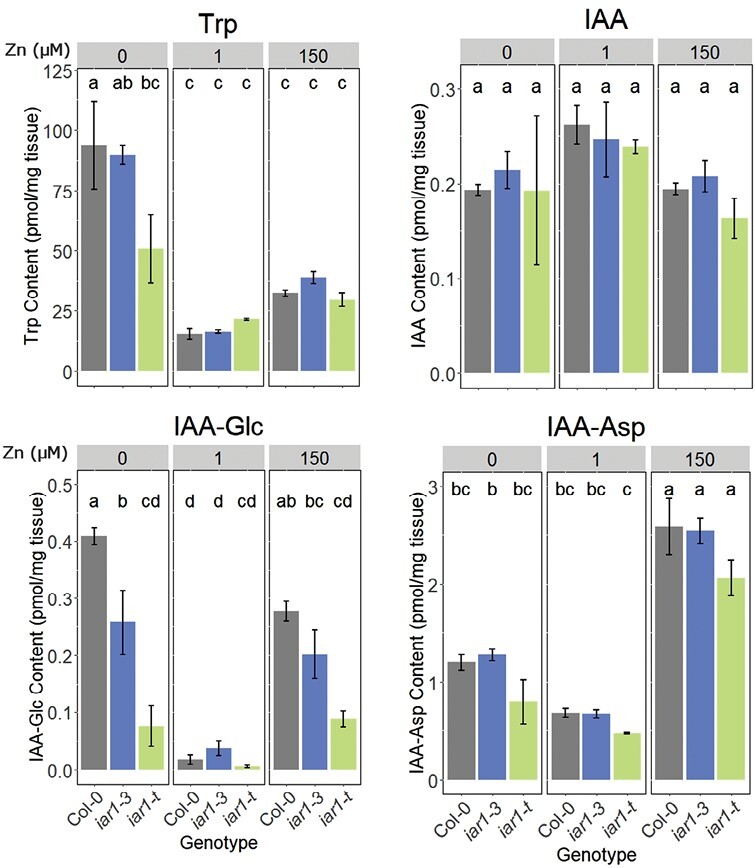
Auxin-related metabolite content. Contents of IAA precursor (Trp), IAA, and IAA conjugates (IAA–Glc, IAA–Asp) were measured from whole Col-0 (grey), *Atiar1-3* (blue), and *Atiar1-t* (green) plants grown for 16 d in Zn-deficient (0 Zn added), Zn control (1 µM), and Zn excess (150 µM) conditions on modified Hoagland’s medium containing EDTA-washed agar. A 100 mg aliquot of fresh tissue was used in each of three biological replicates. Lower case lettering indicates statistically significant differences between groups (labelled sequentially from ‘a’ in order of estimated mean) as calculated using ANOVA utilizing Tukey’s method for *P*-value adjustment (Tukey, 1949) for nine groups with a *P*-value cut-off of 0.05. For analysis of IAN and oxIAA levels, see Supplementary Fig. S3.

In wild-type (Col-0) plants, Zn-deficient conditions induced dramatic increases in Trp and IAA–Glc concentrations (Fig. 3). These results align with findings that Trp levels increase in Zn deficiency as a result of reduced protein synthesis (Cakmak *et al*., 1989). By contrast, under Zn excess, the largest changes were the increases in IAA–Glc and IAA–Asp concentrations. Interestingly, IAA-Glc shows an increase in Zn-deficient and Zn-excess conditions in both Col-0 and *Atiar1-3* plants. However, no significant increase in IAA–Glc was seen in *Atiar1-t* mutants, and the levels in Zn-deficient conditions were less in *Atiar1-3* and *Atiar1-t* genotypes than in Col-0 plants. This suggests that the predicted disruption of IAA–amino acid hydrolysis in *Atiar1* plants leads to compensatory reductions in IAA–Glc pools under high and low Zn conditions. Genotype-specific changes in the levels of oxIAA and IAN were also observed between Zn-excess and Zn-deficient conditions, which further suggests the sensitivity of wider auxin metabolism to Zn and *Atiar1* functionality (Supplementary Fig. S3).

Despite these changes in IAA precursors and conjugates, the concentration of IAA in whole-plant samples remained stable across Zn conditions and genotypes (Fig. 3). As this analysis measured whole-plant IAA levels, it remains possible that IAA distribution or IAA levels at a different developmental stage may be perturbed in Zn- and genotype-specific manners. Since auxin metabolism disturbances in *Atiar1* plants and the activity of IAA–amino acid amidohydrolases are Zn dependent, the Zn dependence of the IAA–Ala sensitivity of *Atiar1* plants was investigated.

### *Atiar1* mutants show an impaired IAA–Ala response and disruption of a novel excess Zn:exogenous auxin interaction

**Fig. 4.**
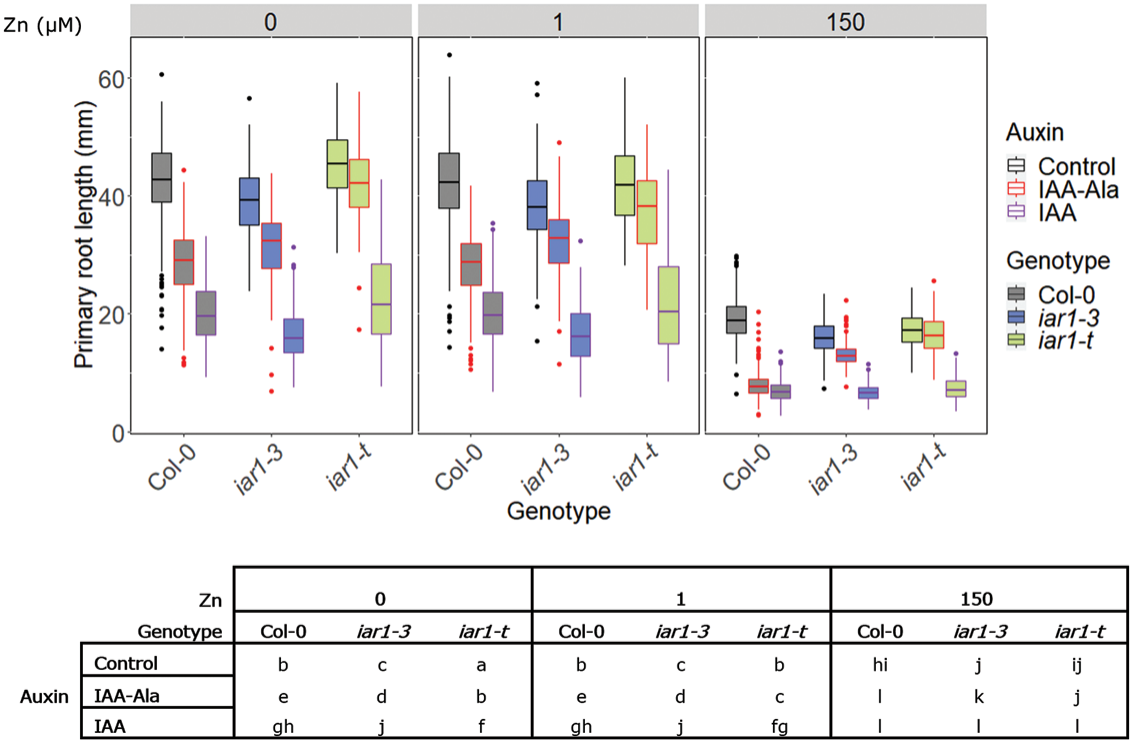
Primary root length of *Atiar1* mutants grown at different Zn concentrations. Primary root length was measured for Col-0 (grey fill), *Atiar1-3* (blue fill), and *Atiar1-t* (green fill) plants 10 d post-germination with medium conditions as in Fig. 3 in control conditions (black outline, with no auxin added), or in the presence of 20 µM IAA–Ala (red outline) or 100 nM IAA (purple outline). For clarity, statistically significant differences between groups (labelled sequentially from ‘a’ in order of estimated mean) are shown in a separate table and were calculated as for Fig. 3 for 27 groups using a *P*-value cut-off of 0.05. For further details on the interaction of genotype, Zn, and auxin condition, see Supplementary Table S8.

The lack of root growth inhibition by IAA–Ala in *Atiar1* plants can be complemented by excess Mn, possibly as a result of activation of IAA–amino acid hydrolases by increased Mn binding (Lasswell *et al*., 2000). However, the IAA–Ala sensitivity of the mutants has not been assessed as a function of different concentrations of Zn, which is hypothesized to inhibit the IAA–amino acid hydrolases (LeClere *et al*., 2002). To test the IAA–Ala sensitivity of the *Atiar1* mutants, primary root lengths 10 d after germination were measured in control medium or in the presence of 20 µM IAA–Ala. Any effect of IAA–Ala could be a function of IAA–Ala uptake, hydrolysis, and subsequent direct or indirect IAA activity. To determine where within this sequence any Zn- and genotype-related phenotypes are acting, the primary root length in medium with 100 nM IAA was also assayed (Fig. 4).

Primary root length has been reported to be inhibited in mutants sensitive to Zn deficiency [*Atbzip19 Atbzip23* double mutants (Assunção *et al*., 2010)] and Zn excess [*Atmtp1* mutants (Kobae *et al*., 2004)]. In Fig. 4 it is shown that in Zn-deficient conditions, with no auxin present, there is a significant reduction in primary root length in *Atiar1-3* mutants relative to the control, although this difference persists across all Zn conditions and so is not Zn specific. On the other hand, the *Atiar1-t* mutant shows increased primary root length only in Zn-deficient conditions. This suggests the two mutants are responding to Zn differently and neither *Atiar1* mutant shows a strong root growth phenotype in Zn-deficient or Zn-excess conditions. Overall, this implies that AtIAR1 is not a major player in Zn homeostasis, further evidenced by chlorophyll and shoot fresh weight analysis which showed a lack of a strong Zn-dependent phenotype (Supplementary Fig. S4).

It is also shown in Fig. 4 that the primary root growth across all Zn conditions in the presence of IAA–Ala is greater in *Atiar1* mutants than in Col-0. The IAA–Ala insensitivity phenotype is more marked in *Atiar1-t* than in *Atiar1-3* mutants, which might suggest that the Atiar1-3 variant protein maintains some partial functionality.

The interaction between genotype and media auxin level was differentially influenced by Zn in the media (*F* statistic 3.8774 and *P*-value 0.0001435: see Supplementary Table S8 for further details). Through conversion of the data in Fig. 4 into percentage primary root growth in Supplementary Fig. S5, it is apparent that for Col-0 the percentage primary root growth is further inhibited by excess Zn compared with control Zn levels in the presence of exogenous IAA–Ala and IAA, an effect which is lost in *Atiar1-3* plants and lessened in *Atiar1-t* plants. As this interaction of Zn, genotype, and auxin condition occurs in IAA-containing media, this three-way interaction occurs post-IAA–Ala hydrolysis.

To determine whether further auxin-related phenotypes show similar Zn dependence of the AtIAR1 and auxin level interaction, shoot hypocotyl length (Supplementary Fig. S6) in media with IAA–Ala and IAA was also measured. The insensitivity to IAA–Ala of *Atiar1* mutants was conserved, as shoot hypocotyls were elongated more in Col-0 than in *Atiar1* mutants in IAA–Ala-containing medium. Additionally, a Zn dependence in the effect of different auxin media on different genotypes was observed (*F* statistic 5.6037, *P*-value <2.2e-16 for the interaction between Zn, genotype, and auxin variables). In excess Zn conditions, shoots of Col-0 were longer in IAA-containing medium than in medium with no auxin—an increase which did not occur in either of the *Atiar1* mutants.

Overall, it seems that two factors are involved in AtIAR1-mediated auxin regulation: conjugate hydrolysis and also a novel Zn excess-mediated effect which appears to act post-hydrolysis in a tissue-specific way. Despite maintaining constant IAA levels across Zn conditions after 16 d, it is possible that changes in auxin levels were seen at different time points or in specific tissues. Therefore, to investigate how auxin-related growth phenotypes might be altered by the disruption of auxin metabolism and altered auxin activity in *Atiar1* mutants, LR density and shoot hypocotyl length during skotomorphogenesis were measured across different Zn conditions.

### *Atiar1* mutants show auxin-related phenotypes

**Fig. 5.**
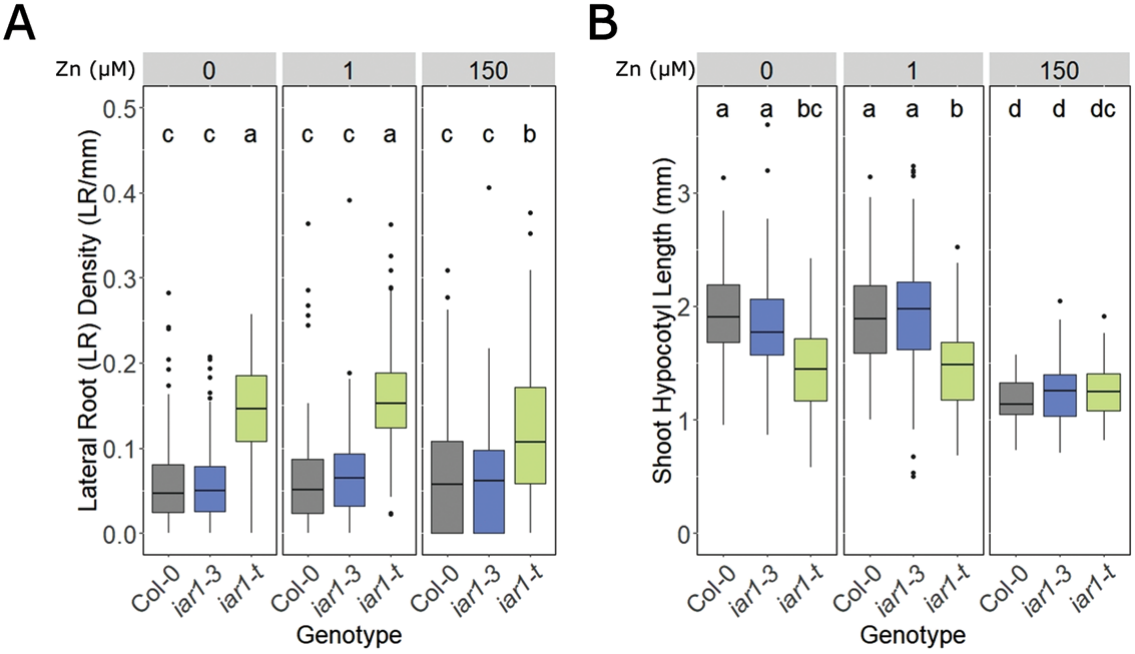
Auxin-related phenotypes are altered in the *Atiar1-t* mutant. (A) Lateral root density or (B) hypocotyl length 10 d after germination was measured for each seedling grown in medium conditions as in Fig. 3. At least 50 plants were measured in each of three biological replicates, with statistically significant differences between groups calculated and displayed as in Fig. 3.

It is shown in Fig. 5A that LR density is selectively increased in *Atiar1-t* mutants and not in *Atiar1-3* mutants, in all Zn conditions. Additionally, LR density is not Zn responsive in wild-type and *Atiar1-3* genotypes whereas in high Zn conditions the LR density of *Atiar1-t* decreases. In Zn deficiency and control Zn conditions, *Atiar1-t* plants showed a reduction in hypocotyl length compared with both Col-0 and *Atiar1-3* plants, further suggesting that Atiar1-3 retains at least partial functionality. For Col-0 and *Atiar1-3* plants at 10 d, Zn excess conditions caused a reduction in shoot hypocotyl length (Fig. 5B). The lack of difference across genotypes in excess Zn conditions could indicate complementation of the *Atiar1-t* phenotype or that hypocotyl length had reached a lower threshold in this condition.

**Fig. 6.**
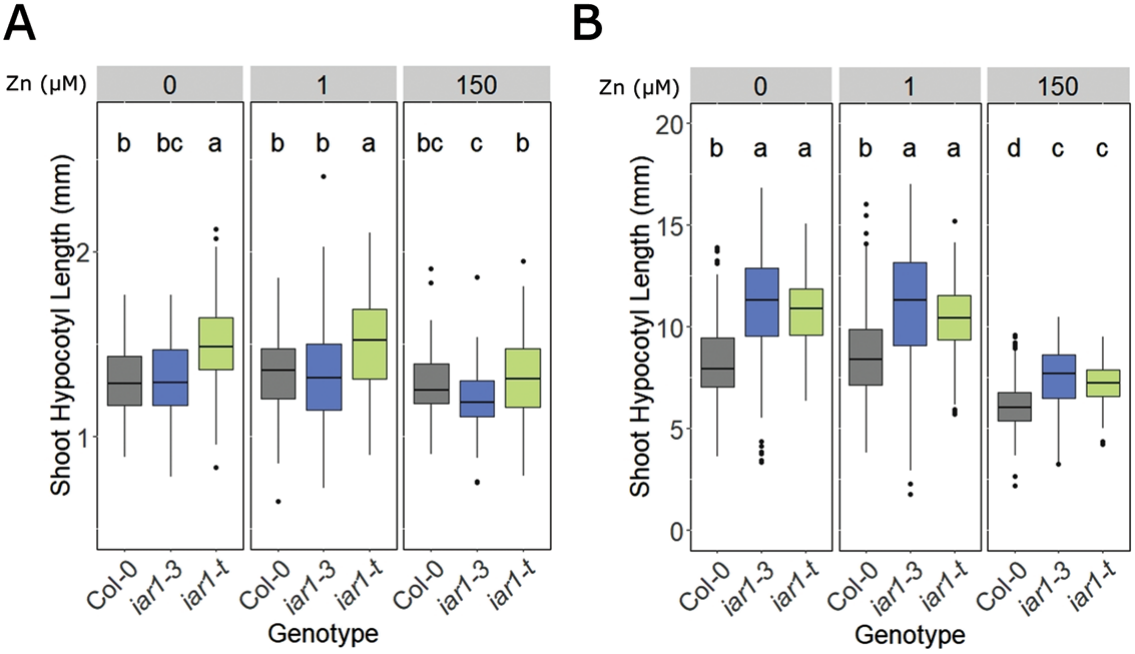
Shoot hypocotyl length in light and dark conditions. Hypocotyl length after growth in light (A) or darkness (B) was measured 5 d after germination for each seedling grown in medium conditions as for Fig. 3. At least 30 plants were measured in each of three biological replicates, with statistically significant differences between groups calculated and displayed as in Fig. 3.

During skotomorphogenesis, auxin plays a key role in hypocotyl elongation through biphasic cell expansion (Du *et al*., 2022). To determine the potential role of Zn and AtIAR1 in hypocotyl elongation in the dark, Col-0 and *Atiar1* mutants were grown in light for 8 h then either kept in the light or transferred to darkness for 4 d, after which hypocotyl lengths were measured (Fig. 6).

After growth for 5 d in light under Zn deficiency and control Zn conditions, *Atiar1-t* plants showed a larger shoot hypocotyl length than the wild type (Fig. 6A). This contrasts with data after 10 d where *Atiar1-t* plants showed smaller hypocotyls (Fig. 5B). Interestingly, the hypocotyl length in skotomorphogenesis was significantly greater compared with Col-0 in both *Atiar1* mutants in all Zn conditions (Fig. 6B). This increased skotomorphogenic hypocotyl length in both *Atiar1* mutants persists across all Zn conditions, including excess Zn conditions where all genotypes show reductions in hypocotyl length.

## Discussion

### AtIAR1 releases Zn ions into the cytosol probably from the secretory system

The complementation of the Zn import mutant strain *zrt1*Δ *zrt2*Δ by *AtIAR1* indicates that AtIAR1 transports Zn. Complementation of this yeast plasma membrane transporter mutant does not necessarily indicate that AtIAR1 is plasma membrane localized in Arabidopsis. For example, the Arabidopsis TPC1 vacuolar membrane protein complements yeast plasma membrane transport mutants (Furuichi *et al*. 2002; Peiter *et al*., 2005).

Our attempts to visualize the intracellular location of AtIAR1 directly with a GFP-expressing construct were not successful because of low expression levels (data not shown). We therefore utilized software prediction packages to predict IAR1 location. The three packages chosen uniformly predicted a secretory system location, although resolution between predicted ER and Golgi localization is not clear (DeepLoc, in addition to the ER, predicted Golgi localization, with a lower probability of 0.62). Therefore, we cannot clearly determine where within the secretory system AtIAR1 may be present.

### *Atiar1* mutants show disruption in auxin metabolism

Disruption of AtIAR1 activity is hypothesized to reduce the hydrolysis and so increase the concentration of reversibly formed IAA–amino acid conjugates by increasing the ER concentration of Zn ions (Lasswell *et al*., 2000). In this study, IAA–Ala was not detectable, but a reduction of reversibly formed IAA–Glc was seen in the *Atiar1-t* mutants. Despite changes in IAA precursors and conjugates across the different Zn conditions and genotypes, the IAA levels did not significantly change.

High levels of auxin promote conjugation to IAA–amino acids and IAA–Glc, as well as reduced synthesis (Suzuki *et al*., 2015). Conversely, ‘low auxin’ metabolic state mutants with the inability to metabolize indole butyric acid, methylated IAA, and IAA–amino acids show compensatory increases in IAA synthesis through TAA/YUCCA induction (Spiess *et al*., 2014). As *Atiar1* mutants are hypothesized to accumulate hydrolysable IAA–amino acids including IAA–Ala but show reduced IAA–Glc levels, it can be hypothesized that the metabolic state of *Atiar1* mutants is of ‘low auxin’. In the ‘low auxin’ model of the *Atiar1-t* mutant, IAA levels are maintained not by the IAA–amino acid hydrolysis which is reduced, but instead by feedback to reduce levels of IAA–Glc. This potential crosstalk between conjugates should be taken into account when analysing mutants with genes responsible for controlling these different conjugation steps. Furthermore, these considerations emphasize the role of IAA conjugates as buffers that smooth IAA concentration changes to establish a required developmental response—albeit in a genotype- and Zn-sensitive manner.

In addition to alteration of auxin metabolism, *Atiar1* mutants also show a disruption of auxin action post-IAA–amino acid hydrolysis in Zn excess conditions. Although the precise mechanisms of Zn excess-induced growth changes are mostly unknown, there is some evidence of altered PIN distribution causing altered auxin accumulation in the root meristem (Zhang *et al*., 2018; Wan *et al*., 2019; Wang *et al*., 2021). Therefore, it is possible that *Atiar1* mutants show altered auxin distribution as well as metabolism.

### Disruption of auxin metabolism in *Atair1* mutants correlates with auxin-related phenotypes

Auxin overproducers such as *AtYUCCA1*-overexpressing plants have increased hypocotyl length in light conditions and decreased hypocotyl length in dark conditions (Zhao *et al*., 2001). *Attmk* loss-of-function mutants show reduced response to exogenous IAA along with reduced cell elongation in shoot and root tissue (Dai *et al*., 2013) and reduced LR number (Huang *et al*., 2019). Similarly, *Atilr1 Atiar3 Atill2* IAA–amino acid hydrolase triple mutants show reduced root length, LR number, and hypocotyl length compared with the wild type (Rampey *et al*., 2004). Previous work has established that the *Atsur1* auxin-overproduction short root phenotype is complemented when crossed with *Atiar1* mutants (Lasswell *et al*., 2000), indicating that *Atiar1* mutants have an effect of reducing auxin action after 10 d. This finding aligns with the suspected low auxin metabolic state of *Atiar1-t* mutants described in this study.

However, the complete loss of function in *Atiar1-t* plants is associated with an unusual combination of auxin-related phenotypes such as increased LR density, a time-dependent shoot hypocotyl length in photomorphogenesis, and increased hypocotyl length in skotomorphogenesis. This resembles neither a typical ‘high auxin’ or a ‘low auxin’ phenotype. These phenotypes are particularly interesting as they occur without any changes in whole-plant IAA levels, suggesting auxin distribution may be perturbed in *Atiar1* mutants due to misregulated auxin conjugates.

Throughout this study, it is evident that *Atiar1-3* and *Atiar1-t* mutations result in different phenotypic severity. As *Atiar1-3* shows intermediate sensitivity between *Atiar1-t* and Col-0 to exogenous IAA–Ala, it is hypothesized that ER Zn levels in *Atiar1-3* are intermediate between those of Col-0 and *Atiar1-t*, and that this leads to milder phenotypes of *Atiar1-3* plants than those of *Atiar1-t* plants, including metabolite levels and developmental changes across different Zn and auxin conditions. Some phenotypes of *Atiar1-3* mutants have, however, been distinct from both *Atiar1-t* and Col-0 plants, which probably relates to the disrupted functionality of the C-terminal region in Atiar1-3.

### AtIAR1 mediates an important intersection of Zn homeostasis and auxin homeostasis in the secretory pathway

Zn transport between the secretory system and the cytosol has previously been shown to be important for both Zn deficiency and the salt stress response (Wang *et al*., 2010; Sinclair *et al*., 2018). Here, we expand the roles of Zn transport between the secretory system and cytosol to auxin metabolic balance and auxin-related growth phenotypes through the activity of AtIAR1. AtIAR1 activity is proposed to release Zn into the cytosol, increasing the response of plants to IAA–Ala through promoting hydrolysis via a mechanism of decreased Zn-mediated inhibition of conjugate hydrolysis within the secretory system. This work on AtIAR1 has also shown the importance of auxin conjugate balance for a variety of physiological auxin responses including LR density and skotomorphogenesis in Arabidopsis.

## Supplementary data

The following supplementary data are available at *JXB* online.

Table S1. Components of modified Hoagland solution.

Table S2. Metabolites measured in this study and sources.

Table S3. Properties of the liquid chromatography column used in this study.

Table S4. Liquid chromatography conditions used in this study.

Table S5. Inlet method for liquid chromatography used in this study.

Table S6. Mass spectrometry conditions used in this study.

Table S7. LC-MS detection parameters for IAA-related metabolites.

Table S8. ANOVA table for primary root length.

Fig. S1. Structures of metabolites measured in this study.

Fig. S2. Atiar1 mutant expression of AtIAR1.

Fig. S3. oxIAA and IAN metabolite content.

Fig. S4. Shoot phenotypes of *Atiar1* mutants grown on different Zn levels.

Fig. S5. Root length percentage change in IAA and IAA–Ala media.

Fig. S6. Shoot hypocotyl length in control, IAA-, and IAA–Ala-containing media.

erad468_suppl_Supplementary_Tables_S1-S8_Figures_S1-S6

## Data Availability

All raw data underlying the results presented in this study are available upon request.

## Abbreviations

Col-0: Columbia ecotype
D_2_-IAA: deuterated IAA
dH_2_O: distilled water
ER: endoplasmic reticulum
GFP: green fluorescent protein
IAA: indole-3-acetic acid
IAN: 3-indoleacetonitrile
LR: lateral root

## References

[CIT0001] Almagro Armenteros JJ , Salvatore M, Emanuelsson O, Winther O, von Heijne H, Elofsson A, Nielsen H. 2019a. Detecting sequence signals in targeting peptides using deep learning. Life Science Alliance 2, e201900429. 31570514 10.26508/lsa.201900429 PMC6769257

[CIT0002] Almagro Armenteros JJ , Tsirigos KD, Sønderby CK, Petersen TN, Winther O, Brunak S, von Heijne G, Nielsen H. 2019b. SignalP 50 improves signal peptide predictions using deep neural networks. Nature Biotechnology 37, 420–423. 10.1038/s41587-019-0036-z 30778233

[CIT0003] Alonso JM , Stepanova AN, Leisse TJ, et al. 2003. Genome-wide insertional mutagenesis of *Arabidopsis thaliana*. Science 301, 653–657. 12893945 10.1126/science.1086391

[CIT0004] Assunção AG , Herrero E, Lin Y-F, et al. 2010. *Arabidopsis thaliana* transcription factors bZIP19 and bZIP23 regulate the adaptation to zinc deficiency. Proceedings of the National Academy of Sciences, USA 107, 10296–10301. 10.1073/pnas.1004788107 PMC2890486 20479230

[CIT0005] Barbez E , Kubeš M, Rolcik J, et al. 2012. A novel putative auxin carrier family regulates intracellular auxin homeostasis in plants. Nature 485, 119–122. 22504182 10.1038/nature11001

[CIT0006] Bartel B , Fink GR. 1995. ILR1, an amidohydrolase that releases active indole-3-acetic acid from conjugates. Science 268, 1745–1748. 7792599 10.1126/science.7792599

[CIT0007] Boerjan W , Cereva MT, Delarue M, Beeckman T, Dewitte W, Bellini C, Caboche M, Van Onckelen H, Van Montagu M, Inzé D. 1995. Superroot, a recessive mutation in Arabidopsis, confers auxin overproduction. The Plant Cell 7, 1405–1419. 8589625 10.1105/tpc.7.9.1405 PMC160963

[CIT0008] Boratyn GM , Camacho C, Cooper PS, et al. 2013. BLAST: a more efficient report with usability improvements. Nucleic Acids Research 41, W29–W33. 23609542 10.1093/nar/gkt282 PMC3692093

[CIT0009] Cakmak I , Marschner H, Bangerth F. 1989. Effect of zinc nutritional status on growth, protein metabolism and levels of indole-3-acetic acid and other phytohormones in bean (*Phaseolus vulgaris* L). Journal of Experimental Botany 40, 405–412.

[CIT0010] Celenza JJ , Grisafi PL, Fink GR. 1995. A pathway for lateral root formation in *Arabidopsis thaliana*. Genes & Development 9, 2131–2142. 7657165 10.1101/gad.9.17.2131

[CIT0011] Cheng Y , Dai X, Zhao Y. 2007. Auxin synthesized by the YUCCA flavin monooxygenases is essential for embryogenesis and leaf formation in Arabidopsis. The Plant Cell 19, 2430–2439. 17704214 10.1105/tpc.107.053009 PMC2002601

[CIT0012] Dai N , Wang W, Patterson SE, Bleecker AR. 2013. The TMK subfamily of receptor-like kinases in Arabidopsis display an essential role in growth and a reduced sensitivity to auxin. PLoS One 8, e60990. 23613767 10.1371/journal.pone.0060990 PMC3628703

[CIT0013] Davies RT , Goetz DH, Lasswell J, Anderson MN, Bartel B. 1999. IAR3 encodes an auxin conjugate hydrolase from Arabidopsis. The Plant Cell 11, 365–376. 10072397 10.1105/tpc.11.3.365 PMC144182

[CIT0014] Desbrosses-Fonrouge A-G , Voigt K, Schröder A, Arrivault S, Thomine S, Krämer U. 2005. *Arabidopsis thaliana* MTP1 is a Zn transporter in the vacuolar membrane which mediates Zn detoxification and drives leaf Zn accumulation. FEBS Letters 579, 4165–4174. 16038907 10.1016/j.febslet.2005.06.046

[CIT0015] Di Mambro R , Svolacchia N, Ioio RD, et al. 2019. The lateral root cap acts as an auxin sink that controls meristem size. Current Biology 29, 1199–1205. 30880016 10.1016/j.cub.2019.02.022

[CIT0016] Dolinsky TJ , Nielsen JE, McCammon JA, Baker NA. 2004. PDB2PQR: an automated pipeline for the setup of Poisson–Boltzmann electrostatics calculations. Nucleic Acids Research 32, W665–W667. 15215472 10.1093/nar/gkh381 PMC441519

[CIT0017] Du M , Daher FB, Liu Y, et al. 2022. Biphasic control of cell expansion by auxin coordinates etiolated seedling development. Science Advances 8, eabj1570. 35020423 10.1126/sciadv.abj1570 PMC8754305

[CIT0018] Dubeaux G , Neveu J, Zelazny E, Vert G. 2018. Metal sensing by the IRT1 transporter-receptor orchestrates its own degradation and plant metal nutrition. Molecular Cell 69, 953–964.e5. 29547723 10.1016/j.molcel.2018.02.009

[CIT0019] Edgar RC. 2004. MUSCLE: multiple sequence alignment with high accuracy and high throughput. Nucleic Acids Research 32, 1792–1797. 15034147 10.1093/nar/gkh340 PMC390337

[CIT0020] Evens NP , Buchner P, Williams LE, Hawkesford MJ. 2017. The role of ZIP transporters and group F bZIP transcription factors in the Zn‐deficiency response of wheat (*Triticum aestivum*). The Plant Journal 92, 291–304. 28771859 10.1111/tpj.13655 PMC5656842

[CIT0021] Franz MC , Simonin A, Graeter S, Hediger M, Kovacs G. 2014. Development of the first fluorescence screening assay for the SLC39A2 zinc transporter. Journal of Biomolecular Screening 19, 909–916. 24619115 10.1177/1087057114526781

[CIT0022] Friml J , Benková E, Blilou I, et al. 2002. AtPIN4 mediates sink-driven auxin gradients and root patterning in Arabidopsis. Cell 108, 661–673. 11893337 10.1016/s0092-8674(02)00656-6

[CIT0023] Fujiwara T , Kawachi M, Sato Y, Mori H, Kutsuna N, Hasezawa S, Maeshima M. 2015. A high molecular mass zinc transporter MTP 12 forms a functional heteromeric complex with MTP 5 in the Golgi in *Arabidopsis thaliana*. The FEBS Journal 282, 1965–1979. 25732056 10.1111/febs.13252

[CIT0024] Furuichi T , Cunningham KW, Muto S. 2002. A putative two pore channel AtTPC1 mediates Ca^2+^ flux in Arabidopsis leaf cells. Plant and Cell Physiology 42, 900–905. 10.1093/pcp/pce145 11577183

[CIT0025] Gietz RD , Woods RA. 2002. Transformation of yeast by lithium acetate/single-stranded carrier DNA/polyethylene glycol method. Methods in Enzymology 350, 87–96. 12073338 10.1016/s0076-6879(02)50957-5

[CIT0026] Guerinot ML. 2000. The ZIP family of metal transporters. Biochimica et Biophysica Acta 1465, 190–198. 10748254 10.1016/s0005-2736(00)00138-3

[CIT0027] Hawkins J , Bodén M. 2006. Detecting and sorting targeting peptides with neural networks and support vector machines. Journal of Bioinformatics and Computational Biology 4, 1–18. 16568539 10.1142/s0219720006001771

[CIT0028] Hoagland DR , Arnon DI. 1938. The water-culture method for growing plants without soil. University of California College of Agriculture Experimental Station Circular 347.

[CIT0029] Huang L , Kirschke CP, Zhang Y, Yu YY. 2005. The *ZIP7* gene (*Slc39a7*) encodes a zinc transporter involved in zinc homeostasis of the Golgi apparatus. Journal of Biological Chemistry 280, 15456–15463. 15705588 10.1074/jbc.M412188200

[CIT0030] Huang R , Zheng R, He J, Zhou Z, Wang J, Xiong Y, Xu T. 2019. Noncanonical auxin signaling regulates cell division pattern during lateral root development. Proceedings of the National Academy of Sciences, USA 116, 21285–21290. 10.1073/pnas.1910916116 PMC6800413 31570617

[CIT0031] Hull AK , Vij R, Celenza JL. 2000. Arabidopsis cytochrome P450s that catalyze the first step of tryptophan-dependent indole-3-acetic acid biosynthesis. Proceedings of the National Academy of Sciences, USA 97, 2379–2384. 10.1073/pnas.040569997 PMC15809 10681464

[CIT0032] Ioio RD , Nakamura K, Moubayidin L, Perilli S, Taniguchi M, Morita MT, Aoyama T, Costantino P, Sabatini C. 2008. A genetic framework for the control of cell division and differentiation in the root meristem. Science 322, 1380–1384. 19039136 10.1126/science.1164147

[CIT0033] Jackson RG , Lim E-K, Li Y, Kowalczyk M, Sandberg G, Hoggett J, Ashford DA, Bowles DJ. 2001. Identification and biochemical characterization of an Arabidopsis indole-3-acetic acid glucosyltransferase. Journal of Biological Chemistry 276, 4350–4356. 11042207 10.1074/jbc.M006185200

[CIT0034] Jeong J , Eide DJ. 2013. The SLC39 family of zinc transporters. Molecular Aspects of Medicine 34, 612–619. 23506894 10.1016/j.mam.2012.05.011 PMC3602797

[CIT0035] Jin S-H , Ma XM, Han P, Wang B, Sun Y-G, Zhang G-Z, Li Y-J, Hou B-K. 2013. UGT74D1 is a novel auxin glycosyltransferase from *Arabidopsis thaliana*. PLoS One 8, e61705. 23613909 10.1371/journal.pone.0061705 PMC3628222

[CIT0036] Jones DT , Taylor WR, Thornton JM. 1992. The rapid generation of mutation data matrices from protein sequences. Computer Applications in the Biosciences 8, 275–282. 1633570 10.1093/bioinformatics/8.3.275

[CIT0037] Jumper J , Evans R, Pritzel A, et al. 2021. Highly accurate protein structure prediction with AlphaFold. Nature 596, 583–589. 34265844 10.1038/s41586-021-03819-2 PMC8371605

[CIT0038] Kobae Y , Uemura T, Sato MH, Ohnishi M, Mimura T, Nakagawa T, Maeshima M. 2004. Zinc transporter of *Arabidopsis thaliana* AtMTP1 is localized to vacuolar membranes and implicated in zinc homeostasis. Plant and Cell Physiology 45, 1749–1758. 15653794 10.1093/pcp/pci015

[CIT0039] Korshunova YO , Eide D, Clark WG, Guerinot ML, Pakrasi HB. 1999. The IRT1 protein from *Arabidopsis thaliana* is a metal transporter with a broad substrate range. Plant Molecular Biology 40, 37–44. 10394943 10.1023/a:1026438615520

[CIT0040] Kumánovics A , Poruk KE, Osborn KA, Ward DM, Kaplan J. 2006. *YKE4* (YIL023C) encodes a bidirectional zinc transporter in the endoplasmic reticulum of *Saccharomyces cerevisiae*. Journal of Biological Chemistry 281, 22566–22574. 16760462 10.1074/jbc.M604730200

[CIT0041] Kumar S , Stecher G, Li M, Knyaz C, Tamura K. 2018. MEGA X: molecular evolutionary genetics analysis across computing platforms. Molecular Biology and Evolution 35, 1547–1549. 29722887 10.1093/molbev/msy096 PMC5967553

[CIT0042] Lasswell J , Rogg LE, Nelson DC, Rongey C, Bartel B. 2000. Cloning and characterization of *IAR1*, a gene required for auxin conjugate sensitivity in Arabidopsis. The Plant Cell 12, 2395–2408. 11148286 10.1105/tpc.12.12.2395 PMC102226

[CIT0043] LeClere S , Tellez R, Rampey RA, Matsuda SPT, Bartel B. 2002. Characterization of a family of IAA–amino acid conjugate hydrolases from Arabidopsis. Journal of Biological Chemistry 277, 20446–20452. 11923288 10.1074/jbc.M111955200

[CIT0044] Li X , Zhang H, Ai Q, Liang G, Yu D. 2016. Two bHLH transcription factors, bHLH34 and bHLH104, regulate iron homeostasis in *Arabidopsis thaliana*. Plant Physiology 170, 2478–2493. 26921305 10.1104/pp.15.01827 PMC4825117

[CIT0045] Liu C , Xu Z, Chua N-H. 1993. Auxin polar transport is essential for the establishment of bilateral symmetry during early plant embryogenesis. The Plant Cell 5, 621–630. 12271078 10.1105/tpc.5.6.621 PMC160300

[CIT0046] Magidin M , Pittman JK, Hirschi KD, Bartel B. 2003. *ILR2*, a novel gene regulating IAA conjugate sensitivity and metal transport in *Arabidopsis thaliana*. The Plant Journal 35, 523–534. 12904214 10.1046/j.1365-313x.2003.01826.x

[CIT0047] Mashiguchi K , Tanaka M, Sakai T, Kasahara H. 2011. The main auxin biosynthesis pathway in Arabidopsis. Proceedings of the National Academy of Sciences, USA 108, 18512–18517. 10.1073/pnas.1108434108 PMC3215075 22025724

[CIT0048] Mateo‐Bonmatí E , Casanova-Sáez R, Šimura J, Ljung K. 2021. Broadening the roles of UDP‐glycosyltransferases in auxin homeostasis and plant development. New Phytologist 232, 642–654. 34289137 10.1111/nph.17633

[CIT0049] Mellor N , Band LR, Pencik A, Owen MR. 2016. Dynamic regulation of auxin oxidase and conjugating enzymes AtDAO1 and GH3 modulates auxin homeostasis. Proceedings of the National Academy of Sciences, USA 113, 11022–11027. 10.1073/pnas.1604458113 PMC5047161 27651495

[CIT0050] Mikkelsen MD , Hansen CH, Wittstock U, Halkier BA. 2000. Cytochrome P450 CYP79B2 from Arabidopsis catalyzes the conversion of tryptophan to indole-3-acetaldoxime, a precursor of indole glucosinolates and indole-3-acetic acid. Journal of Biological Chemistry 275, 33712–33717. 10922360 10.1074/jbc.M001667200

[CIT0051] Milner MJ , Seamon J, Craft E, Kochian LV. 2013. Transport properties of members of the ZIP family in plants and their role in Zn and Mn homeostasis. Journal of Experimental Botany 64, 369–381. 23264639 10.1093/jxb/ers315 PMC3528025

[CIT0052] Mravec J , Skůpa P, Bailly K, et al. 2009. Subcellular homeostasis of phytohormone auxin is mediated by the ER-localized PIN5 transporter. Nature 459, 1136–1140. 19506555 10.1038/nature08066

[CIT0053] Normanly J , Grisafi P, Fink GR, Bartel B. 1997. Arabidopsis mutants resistant to the auxin effects of indole-3-acetonitrile are defective in the nitrilase encoded by the NIT1 gene. The Plant Cell 9, 1781–1790. 9368415 10.1105/tpc.9.10.1781 PMC157021

[CIT0054] Okada K , Ueda J, Komaki MK, Bell CJ, Shimura Y. 1991. Requirement of the auxin polar transport system in early stages of Arabidopsis floral bud formation. The Plant Cell 3, 677–684. 12324609 10.1105/tpc.3.7.677 PMC160035

[CIT0055] Östin A , Kowalyczk M, Bhalerao RP, Sandberg G. 1998. Metabolism of indole-3-acetic acid in Arabidopsis. Plant Physiology 118, 285–296. 9733548 10.1104/pp.118.1.285 PMC34867

[CIT0056] Peiter E , Maathuis FJM, Mills LN, Knight H, Pelloux J, Hetherington AM, Sanders D. 2005. The vacuolar Ca^2+^-activated channel TPC1 regulates germination and stomatal movement. Nature 434, 404–408. 15772667 10.1038/nature03381

[CIT0057] Podar D , Scherer J, Noordally Z, Herzyk P, Nies D, Sanders D. 2012. Metal selectivity determinants in a family of transition metal transporters. Journal of Biological Chemistry 287, 3185–3196. 22139846 10.1074/jbc.M111.305649 PMC3270973

[CIT0058] Porra R , Thompson W, Kriedemann P. 1989. Determination of accurate extinction coefficients and simultaneous equations for assaying chlorophylls a and b extracted with four different solvents: verification of the concentration of chlorophyll standards by atomic absorption spectroscopy. Biochimica et Biophysica Acta 975, 384–394.

[CIT0059] Qin G , Gu Y, Zhao Y, Ma Z, Shi G, Yang Y, Pichersky E, Chen H, Liu M, Chen Z, Qu L-J. 2005. An indole-3-acetic acid carboxyl methyltransferase regulates Arabidopsis leaf development. The Plant Cell 17, 2693–2704. 16169896 10.1105/tpc.105.034959 PMC1242266

[CIT0060] Rampey RA , Baldridge MT, Farrow DC, Bay SN, Bartel B. 2013. Compensatory mutations in predicted metal transporters modulate auxin conjugate responsiveness in Arabidopsis. G3 3, 131–141. 23316445 10.1534/g3.112.004655 PMC3538338

[CIT0061] Rampey RA , LeClere S, Kowalczyk M, Ljung K, Sandberg G, Bartel B. 2004. A family of auxin-conjugate hydrolases that contributes to free indole-3-acetic acid levels during Arabidopsis germination. Plant Physiology 135, 978–988. 15155875 10.1104/pp.104.039677 PMC514132

[CIT0062] Rampey RA , Woodward AW, Hobbs BN, Tierney MP, Lahner B, Salt DE, Bartel B. 2006. An Arabidopsis basic helix–loop–helix leucine zipper protein modulates metal homeostasis and auxin conjugate responsiveness. Genetics 174, 1841–1857. 17028341 10.1534/genetics.106.061044 PMC1698629

[CIT0063] Reinhardt D , Mandel T, Kuhlemeier C. 2000. Auxin regulates the initiation and radial position of plant lateral organs. The Plant Cell 12, 507–518. 10760240 10.1105/tpc.12.4.507 PMC139849

[CIT0064] Sanchez Carranza AP , Singh A, Steinberger K, Panigraphi K, Palme K, Dovzhenko A, Dal Bosco C. 2016. Hydrolases of the ILR1-like family of *Arabidopsis thaliana* modulate auxin response by regulating auxin homeostasis in the endoplasmic reticulum. Scientific Reports 6, 24212. 27063913 10.1038/srep24212 PMC4827090

[CIT0065] Schiavone FM , Cooke TJ. 1987. Unusual patterns of somatic embryogenesis in the domesticated carrot: developmental effects of exogenous auxins and auxin transport inhibitors. Cell Differentiation 21, 53–62. 3607884 10.1016/0045-6039(87)90448-9

[CIT0066] Simon S , Skůpa P, Viaene T, et al. 2016. PIN6 auxin transporter at endoplasmic reticulum and plasma membrane mediates auxin homeostasis and organogenesis in Arabidopsis. New Phytologist 211, 65–74. 27240710 10.1111/nph.14019

[CIT0067] Sinclair SA , Senger T, Talke IN, Cobbett CS, Haydon MJ, Krämer U. 2018. Systemic upregulation of MTP2-and HMA2-mediated Zn partitioning to the shoot supplements local Zn deficiency responses. The Plant Cell 30, 2463–2479. 30150315 10.1105/tpc.18.00207 PMC6241274

[CIT0068] Spiess GM , Hausman A, Yu P, Cohen JD, Rampey RA, Zolman BK. 2014. Auxin input pathway disruptions are mitigated by changes in auxin biosynthetic gene expression in Arabidopsis. Plant Physiology 165, 1092–1104. 24891612 10.1104/pp.114.236026 PMC4081324

[CIT0069] Staswick PE , Serban B, Rowe M, Tiryaki I, Maldonado MT, Maldonado MC, Suza W. 2005. Characterization of an Arabidopsis enzyme family that conjugates amino acids to indole-3-acetic acid. The Plant Cell 17, 616–627. 15659623 10.1105/tpc.104.026690 PMC548830

[CIT0070] Stepanova AN , Robertson-Hoyt J, Yun J, Schlereth A, Jürgens G, Alonso JM. 2008. TAA1-mediated auxin biosynthesis is essential for hormone crosstalk and plant development. Cell 133, 177–191. 18394997 10.1016/j.cell.2008.01.047

[CIT0071] Stepanova AN , Yun J, Robles LM, Novak O, He W, Guo H, Ljung K, Alonso JM. 2011. The Arabidopsis YUCCA1 flavin monooxygenase functions in the indole-3-pyruvic acid branch of auxin biosynthesis. The Plant Cell 23, 3961–3973. 22108406 10.1105/tpc.111.088047 PMC3246335

[CIT0072] Sugahara K , Kitao K, Yamagaki T, Koyama T. 2020. Practical optimization of liquid chromatography/mass spectrometry conditions and pretreatment methods toward the sensitive quantification of auxin in plants. Rapid Communications in Mass Spectrometry 34, e8625. 31658390 10.1002/rcm.8625

[CIT0073] Suzuki M , Yamazaki C, Mitsui M, Kakei Y, Mitani Y, Nakamura A, Ishii T, Soeno K, Shimada Y. 2015. Transcriptional feedback regulation of YUCCA genes in response to auxin levels in Arabidopsis. Plant Cell Reports 34, 1343–1352. 25903543 10.1007/s00299-015-1791-z

[CIT0074] Taylor KM , Morgan HE, Johnson A, Nicholson RI. 2004. Structure–function analysis of HKE4, a member of the new LIV-1 subfamily of zinc transporters. The Biochemical Journal 377, 131–139. 14525538 10.1042/BJ20031183 PMC1223853

[CIT0075] Thumuluri V , Armenteros JJA, Johansen AR, Nielsen H, Winther O. 2022. DeepLoc 20: multi-label subcellular localization prediction using protein language models. Nucleic Acids Research 50, W228–W234. 35489069 10.1093/nar/gkac278 PMC9252801

[CIT0076] Tissot N , Robe K, Gao F, et al. 2019. Transcriptional integration of the responses to iron availability in Arabidopsis by the bHLH factor ILR3. New Phytologist 223, 1433–1446. 30773647 10.1111/nph.15753

[CIT0077] Tsirigos KD , Peters C, Shu N, Käll L, Elofsson A. 2015. The TOPCONS web server for consensus prediction of membrane protein topology and signal peptides. Nucleic Acids Research 43, W401–W407. 25969446 10.1093/nar/gkv485 PMC4489233

[CIT0078] Tukey JW. 1949. Comparing individual means in the analysis of variance. Biometrics 5, 99–114. 18151955

[CIT0079] Vernoux T , Brunoud G, Farcot E, et al. 2011. The auxin signalling network translates dynamic input into robust patterning at the shoot apex. Molecular Systems Biology 7, 508. 21734647 10.1038/msb.2011.39 PMC3167386

[CIT0080] Wan J , Wang R, Wang R, Ju Q, Wang Y, Xu J. 2019. Comparative physiological and transcriptomic analyses reveal the toxic effects of ZnO nanoparticles on plant growth. Environmental Science & Technology 53, 4235–4244. 30871319 10.1021/acs.est.8b06641

[CIT0081] Wang J , Moeen-ud-din M, Yang S. 2021. Dose-dependent responses of *Arabidopsis thaliana* to zinc are mediated by auxin homeostasis and transport. Environmental and Experimental Botany 189, 104554.

[CIT0082] Wang M , Xu Q, Yu J, Yuan M. 2010. The putative Arabidopsis zinc transporter ZTP29 is involved in the response to salt stress. Plant Molecular Biology 73, 467–479. 20358261 10.1007/s11103-010-9633-4

[CIT0083] Wiuf A , Steffen JH, Becares ER, Grønberg C, Mahato DR, Rasmussen SGF, Andersson M, Croll T, Gotfryd K, Gourdon P. 2022. The two-domain elevator-type mechanism of zinc-transporting ZIP proteins. Science Advances 8, eabn4331. 35857505 10.1126/sciadv.abn4331 PMC9278863

[CIT0084] Won C , Shen X, Mashiguchi K, Zhao Y. 2011. Conversion of tryptophan to indole-3-acetic acid by TRYPTOPHAN AMINOTRANSFERASES OF ARABIDOPSIS and YUCCAs in Arabidopsis. Proceedings of the National Academy of Sciences, USA 108, 18518–18523. 10.1073/pnas.1108436108 PMC3215067 22025721

[CIT0085] Yang Y , Xu R, Ma C-J, Vlot AC, Klessig DF, Pichersky E. 2008. Inactive methyl indole-3-acetic acid ester can be hydrolyzed and activated by several esterases belonging to the AtMES esterase family of Arabidopsis. Plant Physiology 147, 1034–1045. 18467465 10.1104/pp.108.118224 PMC2442527

[CIT0086] Zhang P , Sun L, Qin J, Wan J, Wang R, Li S, Xu J. 2018. cGMP is involved in Zn tolerance through the modulation of auxin redistribution in root tips. Environmental and Experimental Botany 147, 22–30.

[CIT0087] Zhao Y , Christensen SK, Fankhauser C, Cashman JR, Cohen JD, Weigel D, Chory J. 2001. A role for flavin monooxygenase-like enzymes in auxin biosynthesis. Science 291, 306–309. 11209081 10.1126/science.291.5502.306

