# Supplementary material for: AtIAR1 is a Zn transporter that regulates auxin metabolism in Arabidopsis thaliana
Source: J Exp Bot. 2023 Nov 21;75(5):1437–50. doi: 10.1093/jxb/erad468 (PMC10901206; doi:10.1093/jxb/erad468)
Supplement: erad468_suppl_Supplementary_Tables_S1-S8_Figures_S1-S6 [file erad468_suppl_supplementary_tables_s1-s8_figures_s1-s6.pdf]

Supplementary Tables

Table S1. Components of modified Hoagland solution

Stock solution	Source	Final Concentration
$Ca(NO_3)_2$	Sigma-Aldrich, C1396	1.5 mM
KH_2PO_4	Sigma-Aldrich, P3786	0.28 mM
$MgSO_4$	Sigma-Aldrich, M2643	0.75 mM
KNO_3	Sigma-Aldrich, 221295	1.25 mM
$CuSO_4$	Sigma-Aldrich, C1297	0.5 µM
$ZnSO_4$	Sigma-Aldrich, Z1001	1 µM
$MnSO_4$	Sigma-Aldrich, M7634	5 µM
H_3BO_3	Sigma-Aldrich, B6768	25 µM
Na_2MoO_4	Sigma-Aldrich, 243655	0.1 µM
KCl	Sigma-Aldrich, 31248	50 µM
MES pH 5.7	Sigma-Aldrich, M8250	3 mM
10 mM FeHBED (10 mM $Fe(NO_3)_3$ + 10.5 mM HBED)	Sigma-Aldrich, 216828 and Strem Chemicals, 35369-53-0	5 µM

Table S2. Metabolites measured in this study and sources

Metabolite	Source
L-Tryptophan (Trp)	Sigma-Aldrich, T0254
3-Indoleacetonitrile (IAN)	Sigma-Aldrich, 129453
3-Indoleacetic acid (IAA)	Sigma-Aldrich, 45533
Indole-3-acetyl b-D-Glucopyranose (IAA-Glc)	Toronto Research Chemicals, I627020
(2S)-2-[[2-(1H-Indol-3-yl)acetyl]amino]butanedioic acid (IAA-Asp)	Astatech, A11375
N-(3-Indolylacetyl)- L -alanine (IAA-Ala)	Sigma-Aldrich, 345911
2-Oxo-2,3-dihydro-1H-indol-3-yl)acetic acid (oxIAA)	Sigma-Aldrich, CDS009127
Indole-3-acetic-2,2-D_2 acid (D_2-IAA)	Sigma-Aldrich, 492817

Table S3. Properties of LC column used in this study

Property of LC column	Value
Internal diameter (mm)	2.1
Length (mm)	100
Stationary phase	C18 with trimethylsilyl endcapping
Solid Support	Organo-silica with ethane cross-linking,
Particle size (µm)	2.6
Pore size (Å)	100

Table S4. LC conditions used in this study

LC condition	Value
Solvent A	0.5% (v/v) formic acid (Supelco, 00940) dissolved in Milli-Q ultrapure water
Solvent B	100% methanol
Column temperature (°C)	40
Flow rate (mL/min)	0.4
Injection volume (µL)	5

Table S5. Inlet method for LC used in this study

Time	Solvent A%	Solvent B%
0	95	5
4	10	90
5	10	90
5.2	95	5
8	STOP	STOP

Table S6. MS conditions used in this study

MS condition	Value
Ionisation mode	Electrospray ionisation
Capillary voltage (kV)	1.5
Cone voltage (V)	20
Source offset (V)	50
Desolvation gas temperature (°C)	500
Desolvation gas flow (L/hr)	1000
Cone gas flow (L/hr)	150
Nebulising gas pressure (bar)	70
Collision gas flow (mL/min)	0.15

Table S7. LC-MS detection parameters for IAA-related metabolites. Serial dilutions of metabolite standards were separated and identified by LC-MS. Retention time window used for detection was the stated retention time ± 0.2 min.

Metabolite	Retention time (min)	Product detection (m/z)	Linear range (nM)	R^2
Trp	1.04	205 >118	5 - 1000	0.9973
IAN	2.28	157 >130	5 - 10000	0.9974
IAA	1.89	176 >130	5 - 1000	0.9929
oxIAA	1.49	192 > 146	1 - 1000	0.9962
IAA-Ala	1.78	247 > 90	0.5 - 500	0.9998
IAA-Asp	1.57	291 > 130	0.5 - 500	0.9999
IAA-Glc	1.53	338 > 130	5 - 1000	0.9991
D_2-IAA	1.89	178 > 132	n/a	n/a

Table S8. Analysis of Variance table for primary root length. Data used to construct Figure 4. Df = degrees of freedom, Sum Sq = sum of squares, Mean Sq = Mean Sum Sq, Pr(>F) = probability of non-significant interaction.

	Df	Sum Sq	Mean Sq	F value	Pr(>F)
Auxin	2	287287	143643	4907.6475	< 2.2e-16
Genotype	2	19620	9810	335.1639	< 2.2e-16
Zn	2	448201	224101	7656.5134	< 2.2e-16
Auxin:Genotype	4	20309	5077	173.4693	< 2.2e-16
Auxin:Zn	4	29028	7257	247.9425	< 2.2e-16
Genotype:Zn	4	5849	1462	49.9576	< 2.2e-16
Auxin:Genotype:Zn	8	908	113	3.8774	0.0001435
Residuals	5516	161449	29		

Supplementary Figures

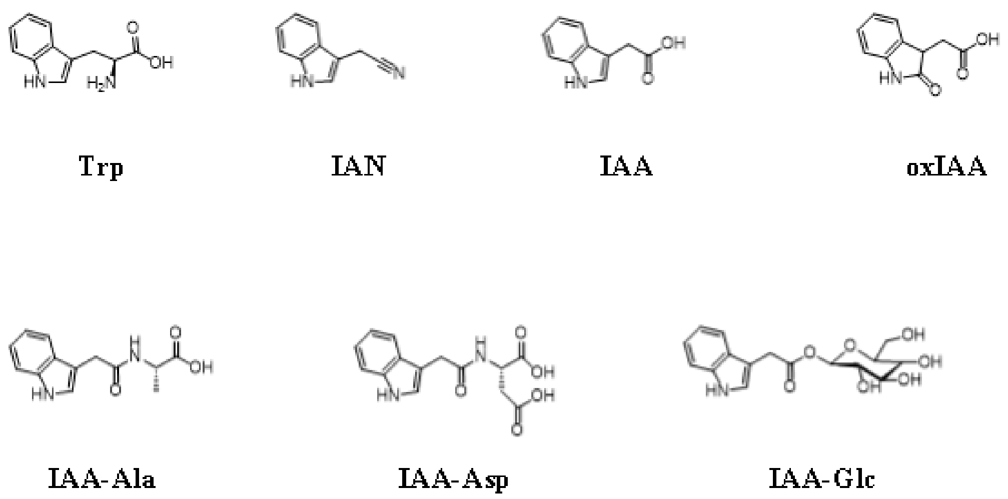

Figure S1 Structures of metabolites measured in this study. Created using ChemDraw 2.0 (PerkinElmer).

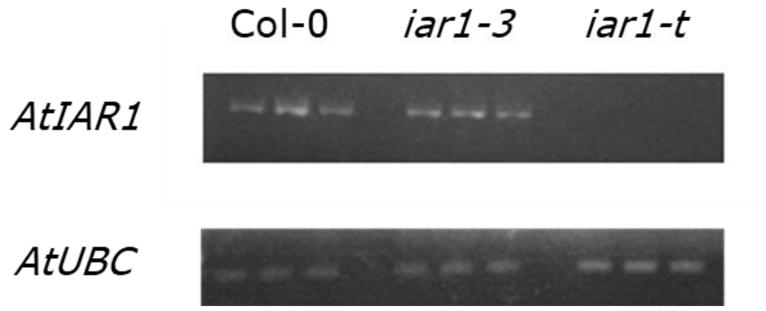

Figure S2 *Atiar1* mutant expression of *AtIAR1*. Amplification of full length *AtIAR1* and a control gene *UBIQUITIN C* (*AtUBC)* fragment from cDNA gathered from three independent Col-0, *Atiar1-3* and *Atiar1-t* samples.

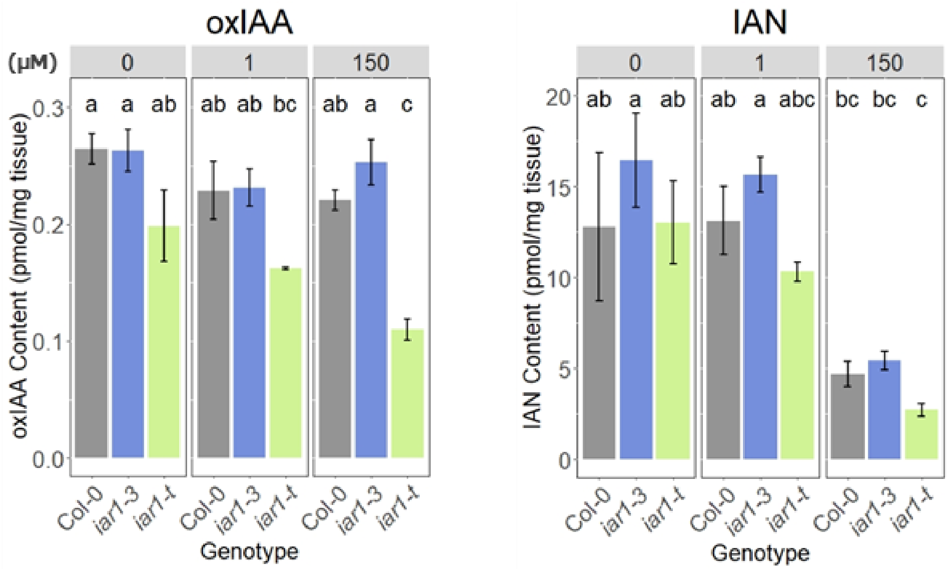

Figure S3 Auxin related metabolite content. Content of oxIAA and IAN measured from whole Col-0 (grey), *Atiar1-3* (blue) and *Atiar1-t* (green) plants grown for 16 days in Zn deficient (0 Zn added), Zn control (1 µM) and Zn excess (150 µM) conditions on modified Hoagland's media containing EDTA-washed agar. 100 mg of fresh tissue was used in each of three biological replicates. Lower case lettering indicates statistically significant differences between groups (labelled sequentially from 'a' in order of estimated mean) as calculated using analysis of variance (ANOVA) using Tukey's method (Tukey, 1949) for p-value adjustment for 9 groups using a p-value cut-off of 0.05.

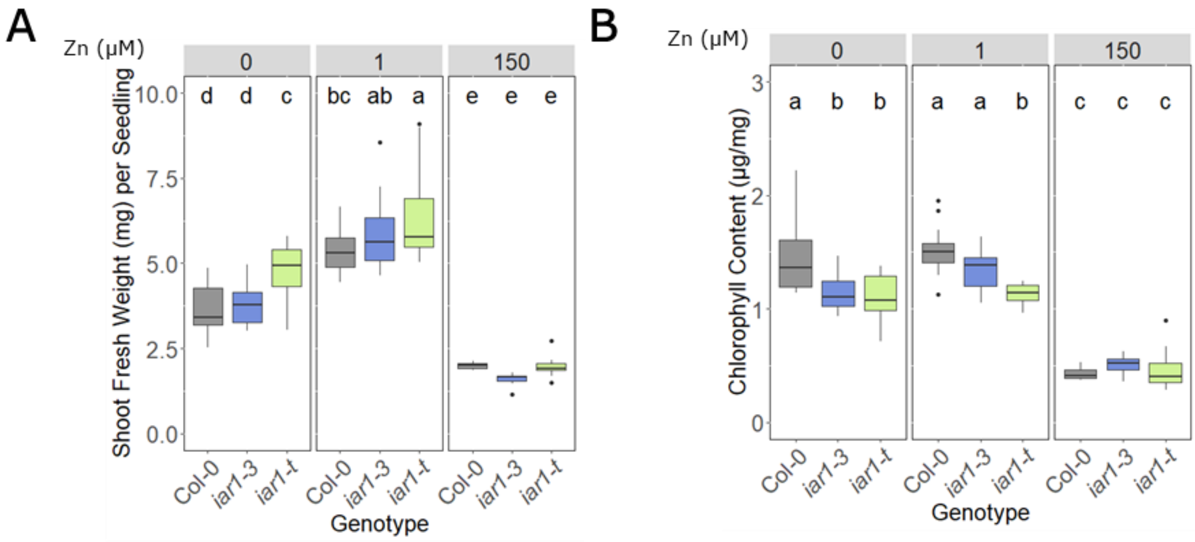

Figure S4 Shoot phenotypes of *Atiar1* mutants grown on different Zn levels. (A) Shoot fresh weight and (B) chlorophyll content was measured from Col-0 (grey), *Atiar1-3* (blue) and *Atiar1-t* (green) plants grown for 16 days in Zn deficient (0 Zn added), Zn control (1 µM) and Zn excess (150 µM) conditions on modified Hoagland's media containing EDTA-washed agar. For each of 3 biological replicates, plant shoot tissue from at least 30 plants was separated into 50 mg pools before measuring. Statistically significant differences between groups were calculated and displayed as in **Figure S3.**

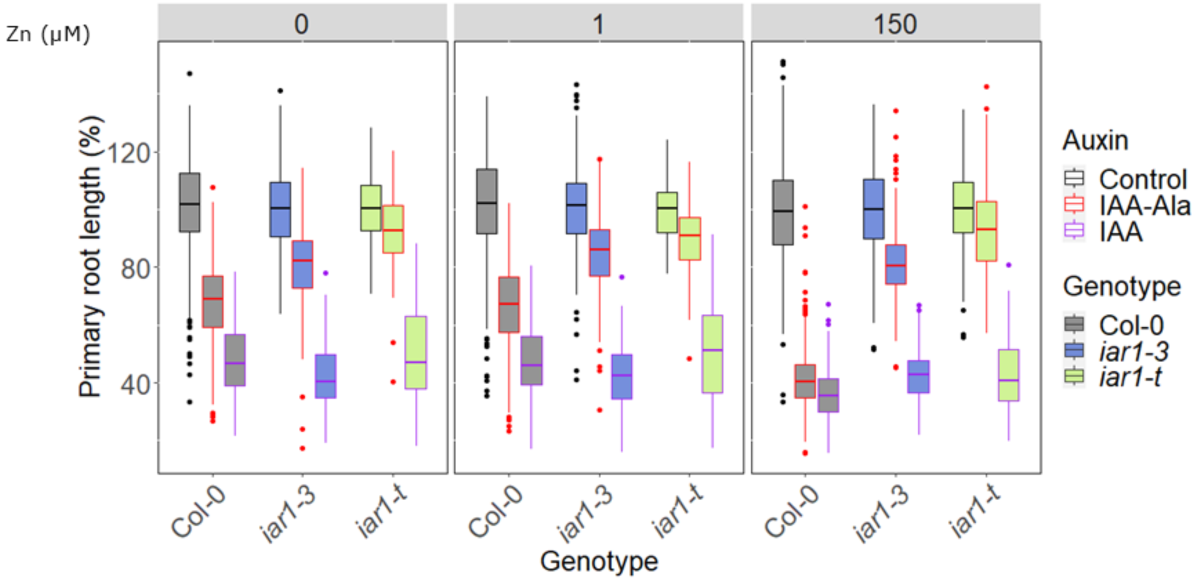

Zn		0			1			150		
	**Genotype**	**Col-0**	***iar1-3***	***iar1-t***	**Col-0**	***iar1-3***	***iar1-t***	**Col-0**	***iar1-3***	***iar1-t***
**Auxin**	**Control**	a	a	a	a	a	a	a	a	a
	**IAA-Ala**	e	d	b	e	cd	bc	h	d	b
	**IAA**	fg	h	f	fg	h	f	i	h	gh

	Df	Sum Sq	Mean Sq	F value	Pr(>F)
Auxin	2	2852738	1426369	7315.636	< 2.2e-16
Genotype	2	145088	72544	372.067	< 2.2e-16
Zn	2	23249	11625	59.621	< 2.2e-16
Auxin:Genotype	4	221009	55252	282.28	< 2.2e-16
Auxin:Zn	4	14563	3641	18.673	2.937e-15
Genotype:Zn	4	34211	8553	43.866	< 2.2e-16
Auxin:Genotype:Zn	8	37877	4735	24.284	< 2.2e-16
Residuals	5516	1075484	195		

Figure S5 Root length percentage change in IAA and IAA-Ala media. Col-0 (grey), *Atiar1*-3 (blue) and *Atiar1-t* (green) plants grown for 10 days in Zn deficient (0 Zn added), Zn control (1 µM) and Zn excess (150 µM) conditions on modified Hoagland's media containing EDTA-washed agar. Percentage change of primary root length in media containing 20 µM IAA-Ala (red outline) or 100 nM IAA (purple outline) compared to control media with no auxin conjugate or auxin added (black outline). Lower case lettering indicates statistically significant differences between groups (labelled sequentially from 'a' in order of estimated mean) as calculated using analysis of variance (ANOVA) using Tukey's method for p-value adjustment for 27 groups (Tukey, 1949) using a p-value cut-off of 0.05, and is listed separately in a table for clarity, alongside the associated analysis of variance table of statistics as for **Table S8**.

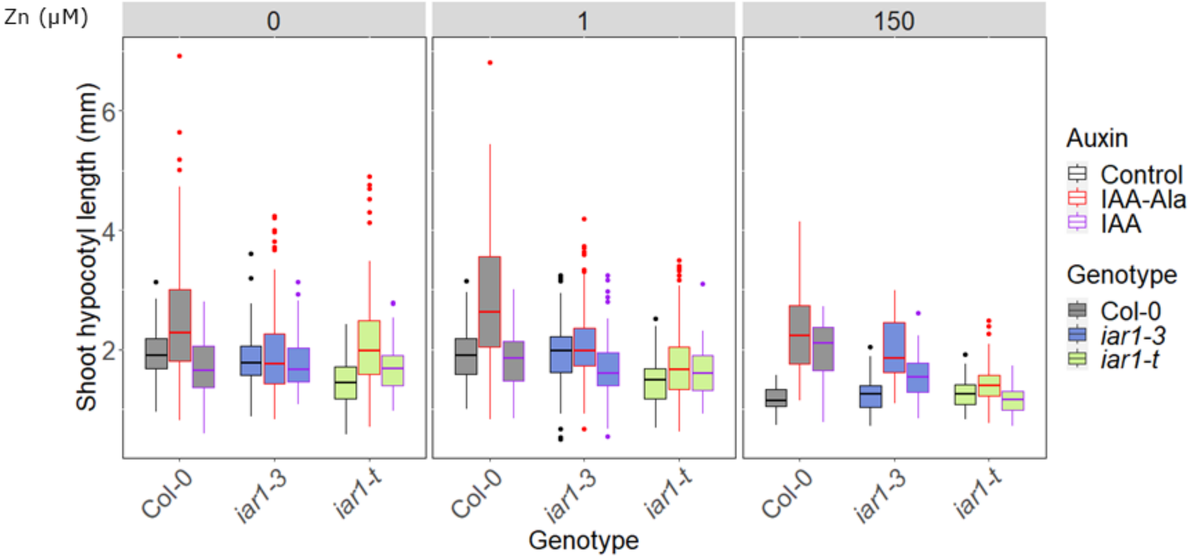

	Zn	0			1			150		
	Genotype	Col-0	*iar1-3*	*iar1-t*	Col-0	*iar1-3*	*iar1-t*	Col-0	*iar1-3*	*iar1-t*
Auxin	Control	cd	cde	ghi	cd	cd	fghi	i	i	hi
	IAA-Ala	b	cde	defg	a	c	def	defg	ghi	ghi
	IAA	defg	defg	hi	cd	defg	defgh	efghi	i	i

	Df	Sum Sq	Mean Sq	F value	Pr(>F)
Auxin	2	98.85	49.426	150.31	< 2.2e-16
Genotype	2	127.46	63.73	193.81	< 2.2e-16
Zn	2	163.65	81.824	248.8364	< 2.2e-16
Auxin:Genotype	4	36.92	9.231	28.0726	< 2.2e-16
Auxin:Zn	4	5.87	1.467	4.46	0.00136
Genotype:Zn	4	24.94	6.235	18.96	2.283e-15
Auxin:Genotype:Zn	8	14.74	1.843	5.6037	< 2.2e-16
Residuals	2443	803.32	0.329		

Figure S6 Shoot hypocotyl length in control, IAA and IAA-Ala containing media. Col-0 (grey fill), *Atiar1*-3 (blue fill) and *Atiar1-t* (green fill) plants grown for 10 days in Zn deficient (0 Zn added), Zn control (1 µM) and Zn excess (150 µM) conditions on modified Hoagland's media containing EDTA-washed agar in control media containing no auxin (black outline as a control), 20 µM IAA-Ala (red outline) or 100 nM IAA (purple outline). Lower case lettering indicates statistically significant differences between groups and is displayed as in **Figure S3** alongside the associated analysis of variance table of statistics as for **Table S8**.
